# Prediction efficiency and incremental processing strategy during spoken language comprehension in autistic children: an eye-tracking study

**DOI:** 10.1186/s13229-025-00674-0

**Published:** 2025-08-04

**Authors:** Zihui Hua, Tianbi Li, Ruoxi Shi, Ran Wei, Li Yi

**Affiliations:** 1 School of Psychological and Cognitive Sciences & Beijing Key Laboratory of Behavior and Mental Health, Peking University, 5 Yiheyuan Road, Beijing, 100871 China; 2 Department of Psychology, School of Education Science, Qingdao University, Qingdao, China; 3 Faculty of Education, University of Macau, Macao, China; 4 Graduate School of Education, Peking University, 5 Yiheyuan Road, Beijing, 100871 China; 5 IDG/McGovern Institute for Brain Research at PKU, Peking University, Beijing, China

**Keywords:** Autism, Children, Prediction, Predictive processing, Incremental processing, Language comprehension, Language processing, Eye tracking

## Abstract

**Background:**

Language difficulties are common in autism, with several theoretical perspectives proposing that difficulties in forming and updating predictions may underlie the cognitive profile of autism. However, research examining prediction in the language domain among autistic children remains limited, with inconsistent findings regarding prediction efficiency and insufficient investigation of how autistic children incrementally integrate multiple semantic elements during language processing. This study addresses these gaps by investigating both prediction efficiency and incremental processing strategy during spoken language comprehension in autistic children compared to neurotypical peers.

**Methods:**

Using the visual world paradigm, we compared 45 autistic children (3–8 years) with 52 age-, gender-, and verbal IQ-matched neurotypical children. Participants viewed arrays containing a target object and three semantically controlled distractors (agent-related, action-related, and unrelated) while listening to subject-verb-object structured sentences. Eye movements were recorded to analyze fixation proportions. We employed cluster-based permutation analysis to identify periods of sustained biased looking, growth curve analysis to compare fixation trajectories, and divergence point analysis to determine the onset timing of predictive looking.

**Results:**

Both groups demonstrated predictions during spoken language comprehension and employed similar incremental processing strategies, showing increased fixations to both target objects and action-related distractors after verb onset despite the latter’s incompatibility with the agent. However, autistic children exhibited reduced prediction efficiency compared to neurotypical peers, evidenced by significantly lower proportions of and slower growth rates in fixations to target objects relative to unrelated distractors, and delayed onset of predictive looking. Reduced prediction efficiency was associated with higher levels of autism symptom severity in the autistic group and increased autistic traits across both groups, with autism-related communication difficulties showing the most robust associations.

**Limitations:**

Our sample included only autistic children without language impairments, limiting generalizability to the broader autism spectrum. The task employed only simple sentence structures in controlled experimental settings, which may not fully capture language processing patterns in naturalistic communication contexts.

**Conclusions:**

While autistic children employ similar incremental processing strategies to neurotypical peers during language comprehension, they demonstrate reduced prediction efficiency. Autism symptom severity and autistic traits varied systematically with prediction efficiency, with autism-related communication difficulties showing the strongest associations. These findings enhance our understanding of language processing mechanisms in autism and suggest that interventions targeting language development might benefit from addressing prediction efficiency, such as providing additional processing time and gradually increasing the complexity of semantic integration tasks.

**Supplementary Information:**

The online version contains supplementary material available at 10.1186/s13229-025-00674-0.

## Background

Autism is a neurodevelopmental condition characterized by persistent difficulties in social communication and interaction, alongside restricted and repetitive patterns of behavior or interests [1]. Several theoretical perspectives propose that difficulties in forming and updating predictions may constitute a fundamental mechanism underlying the cognitive profile of autism [2-5]. However, research examining prediction in the language domain among autistic children remains limited, with inconsistent findings regarding their prediction efficiency. This gap is particularly significant given that autistic children commonly demonstrate language difficulties and delayed language development, though both receptive and expressive language abilities vary considerably across individuals in this population [1, 6, 7]. Further exploration of predictive processing in autistic children is needed to better understand their language difficulties. Furthermore, the specific strategy through which predictions are incrementally formed and updated (i.e., incremental processing strategy) during language comprehension remains largely unexplored in this population. Given that real-world language comprehension requires continuous integration of unfolding linguistic information, understanding the incremental processing strategy adopted by autistic children is essential for developing a comprehensive account of their language processing mechanisms and the challenges they may face in natural communication contexts. Therefore, this study aims to investigate how autistic children predict upcoming linguistic information during spoken language comprehension by both characterizing their incremental processing strategy and examining their prediction efficiency.

Language comprehension is a complex and dynamic process in which listeners not only decode language input they have already heard but also predict upcoming information [8, 9]. During real-time language comprehension, individuals activate and utilize existing knowledge and contextual cues to predict forthcoming linguistic information [9, 10]. This predictive mechanism significantly enhances language processing efficiency, enabling children to allocate additional cognitive resources to other information processing demands, thereby playing a crucial role in language development [11, 12]. Among the methodological approaches employed to investigate prediction in language processing, the visual world paradigm has been widely implemented [10, 13]. This paradigm records participants’ eye movement patterns as they listen to linguistic stimuli while simultaneously viewing visual arrays, thereby enabling inference of real-time language processing through the analysis of eye movement behaviors [13, 14]. Studies utilizing this paradigm frequently contrast biased versus neutral verbs (e.g., “eat the cake” versus “move the cake”) and measure predictive eye movements toward potential objects [8, 11, 15-18]. In such biased-verb paradigms, neurotypical (NT) participants demonstrate faster and more frequent fixations to semantically appropriate objects (e.g., cake) upon hearing constraining verbs (e.g., “eat”) than to neutral verbs (e.g., “move”) [8]. Research employing this paradigm has revealed that from 2 to 3 years of age, NT children listening to simple subject-verb-object (SVO) structured sentences exhibit predictive fixations toward images corresponding to objects before actually hearing object words, demonstrating the ability to utilize single semantic cues to predict subsequent information [19, 20]. Moreover, prediction efficiency, as measured by fixation proportions to the target object, has been found to be correlated with language proficiency in NT children. In 2-year-old NT children, prediction efficiency correlates significantly with vocabulary size [20], and in NT children aged 3–10 years, prediction efficiency correlates with both receptive and expressive vocabulary skills [19, 21], highlighting the crucial role of prediction in language development.

Various theoretical frameworks have elucidated the cognitive characteristics of autism through the lens of prediction [2, 3, 5]. Gomot and Wicker [2] first proposed that autistic individuals experience impairments in predictive ability, suggesting that these difficulties might represent a fundamental mechanism underlying core autism features and potentially hinder adaptation to changing environments or unexpected events. Sinha et al. [3] further proposed the predictive impairment in autism hypothesis, emphasizing the difficulties autistic individuals encounter in establishing predictive relationships between phenomena and their environmental context. For example, the social communication challenges experienced by autistic individuals may stem from difficulties in detecting probabilistic relationships between linguistic or social cues and their likely responses, thereby rendering social interactions unpredictable and overwhelming [3]. From a Bayesian perspective, Pellicano and Burr [22] proposed that autistic individuals may have attenuated or less precise prior expectations, leading to perception that is less influenced by top-down predictions. Van de Cruys et al. [23] additionally proposed that autistic individuals might experience difficulties in processing and calibrating prediction errors, a capacity crucial for prediction efficiency. A recent systematic review has provided comprehensive evidence supporting these theoretical perspectives, demonstrating distinct differences in both predictive learning and predictive response in autism [5].

Given the crucial role of prediction in language comprehension, these theoretical perspectives suggest that autistic children might demonstrate distinctive prediction efficiency during language processing. However, empirical findings in this domain remain inconsistent. Several studies have indicated that autistic children aged 5 to 9 years can utilize verb semantics for prediction during language comprehension, demonstrating prediction efficiency comparable to that of age- or language ability-matched NT children [15, 16]. Brennan et al. [24] found no significant differences between autistic and age-matched NT children in predictive sentence parsing efficiency via magnetoencephalography technology. On the other hand, Zhou et al. [18] reported that although 5-year-old autistic children exhibited verb-based predictive eye movements during sentence comprehension as age- or language ability-matched NT children did, their fixation proportions to target objects were significantly lower than those of age-matched NT children. Prescott et al. [17] reported that 3- to 4-year-old autistic children demonstrated reduced differentiation between conditions with constraining information and those without, compared to language ability-matched NT children, suggesting diminished utilization of semantic information during prediction. Furthermore, Bavin, Prendergast et al. [25] found that when integrating multiple constraining elements (adjectives, nouns) in sentences to predict targets, 5- to 9-year-old autistic children exhibited slower prediction and reduced proportions of target fixations than age-matched NT children. It should be noted that these studies varied in their matching criteria, with some matching on language ability or verbal IQ in addition to or instead of age [16, 17], some matching on age but not verbal measures [15, 24, 25], and some including both age-matched and language ability-matched comparison groups [18]. Collectively, existing evidence suggests that while autistic children maintain prediction abilities during language processing, they may exhibit distinctive patterns regarding prediction efficiency, particularly while integrating multiple semantic cues.

Despite these valuable insights into autistic children’s prediction abilities during language processing, existing research has predominantly focused on single-element-based prediction (i.e., the use of single semantic elements such as a biased verb to predict subsequent information), as demonstrated in classic biased-verb paradigms [11, 15-18]. However, in real-world contexts, linguistic input typically contains multiple semantic elements that unfold sequentially over time. As these semantic elements are revealed, listeners must continuously integrate new information with previously processed content, dynamically adjusting and updating their earlier predictions in real-time. This reflects the inherently *incremental* nature of language processing [10, 19]. Understanding this incremental process is particularly important because it better captures the demands of authentic communication situations where predictions are not based on isolated cues but on continuously evolving contextual information.

**Fig. 1:**
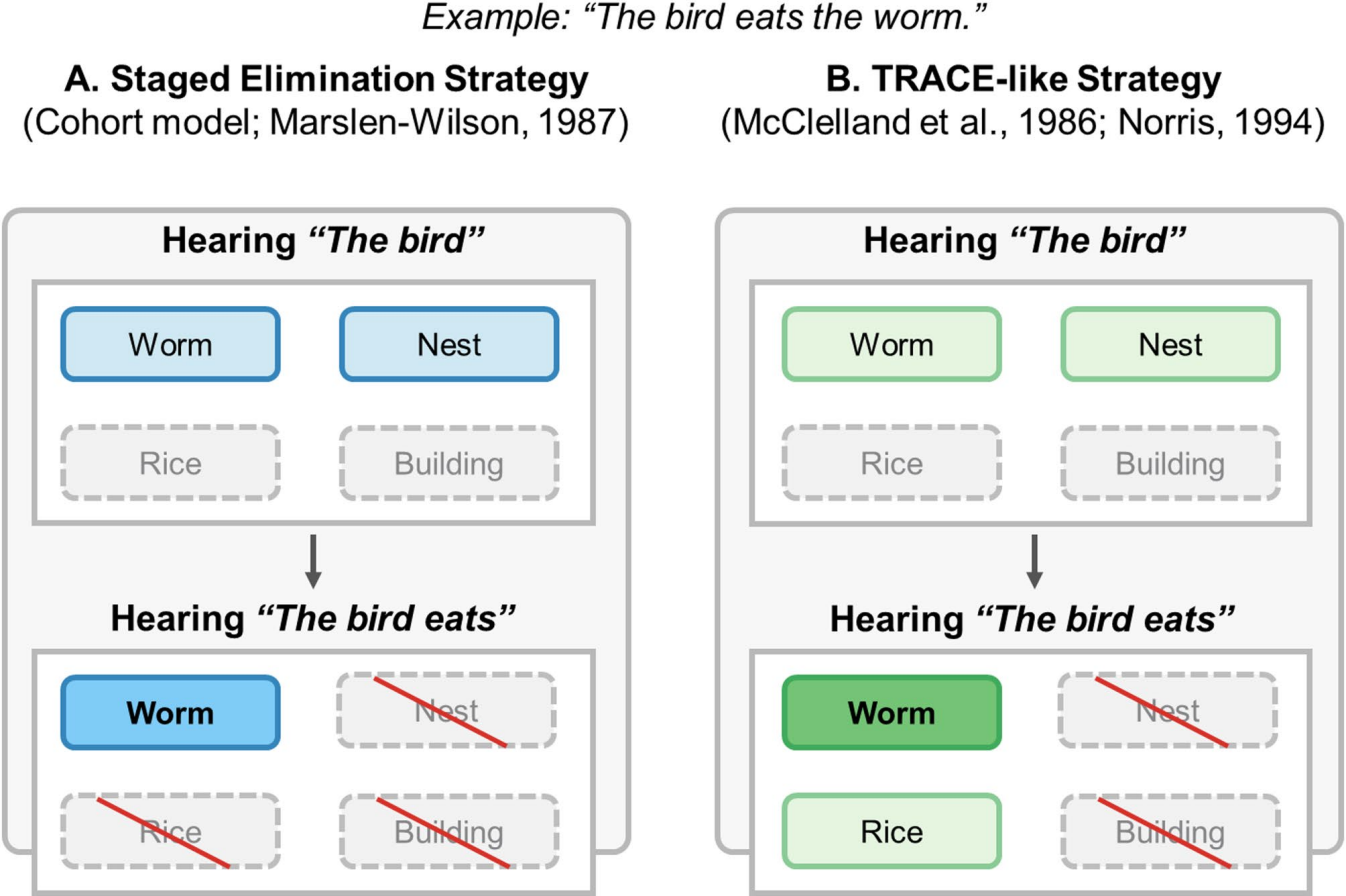
Comparison of incremental processing strategies. In the case of SVO-structured sentence processing, after hearing the agent, listeners would initially activate items semantically related to the agent (worm and nest). Then, if listeners employ the staged elimination strategy, upon hearing the action, they would eliminate items that do not meet both constraints, leaving only the activation of the target that matches both the agent and action (worm). In contrast, if listeners employ the TRACE-like strategy, after hearing the action, both the target (worm) and the action-related item (rice) become active, even if not fully compatible with all constraints

During language processing, two potential incremental processing strategies have been identified [19, 26, 27]. One strategy involved staged elimination, analogous to the Cohort model of word recognition [28]: As sentences unfold, subsequent information primarily serves to eliminate incompatible candidates until a single option remains activated. In contrast, the TRACE-like strategy, paralleling the TRACE model of speech perception [26] and the Merge/Shortlist model [29], suggests that as sentence unfolds, subsequent information not only eliminates initially activated inappropriate options but also activates novel options consistent with current information, even if incompatible with earlier information (see Fig. 1 for comparison between these two incremental processing strategies). Empirical evidence indicates that NT children and adults tend to adopt a TRACE-like model during spoken sentence comprehension [19, 30]. However, to our knowledge, research has not examined whether autistic children employ similar incremental processing strategies. Investigating the incremental processing strategy of autistic children is crucial as it provides a better understanding of their prediction mechanism during language comprehension and may inform interventions targeting language development and communication skills [11].

In summary, although existing research has provided valuable insights into prediction during language processing in autistic children, findings regarding their prediction efficiency demonstrate inconsistency, necessitating additional investigation. Furthermore, existing research has predominantly focused on single-element-based prediction, whereas insufficient attention has been given to how autistic children incrementally integrate multiple semantic elements and update their prediction as sentences unfold (i.e., incremental processing strategy). This study aims to address these gaps by examining both prediction efficiency and incremental processing strategy in autistic children. This dual focus will contribute meaningfully to our understanding of language comprehension mechanisms in autism and potentially inform interventions targeting language development in this population.

Drawing from Borovsky et al.‘s [19] experimental design, we employ the visual world paradigm in which participants listen to simple SVO-structured sentences while viewing visual scenes containing a target object and three semantically controlled distractors: an agent-related distractor, an action-related distractor, and an unrelated distractor. Compared to classic biased-verb paradigms that examine single-element-based prediction [11, 17, 18], this design examines predictions based on dual linguistic elements, enabling differentiation between the two potential incremental processing strategies and may provide deeper insights into the incremental and dynamic nature of language processing in autistic children.

Our specific research questions are threefold: what incremental processing strategy autistic children employ during real-time spoken sentence comprehension, how their prediction efficiency differs from that of NT peers, and whether prediction efficiency varies with autism symptom severity and autistic traits. Based on existing literature demonstrating comparable patterns of semantic processing between autistic and NT children [16, 31, 32] and guided by the prediction impairment in autism hypothesis [2, 3, 23], we formulated three main hypotheses: First, both autistic children and NT children would demonstrate the ability to integrate semantic information from the subject (agent) and verb (action) to predict the upcoming object (patient), employing a TRACE-like incremental processing strategy rather than a staged elimination approach. Specifically, we expected both groups to show increased fixations not only to the target object but also to action-related distractors after hearing the verb, despite the action-related distractors being incompatible with the agent. Second, we hypothesized that autistic children would demonstrate reduced prediction efficiency compared to NT children. We expected this to manifest as reduced fixation proportions to target objects relative to unrelated distractors and delayed onset of predictive looking toward target objects after hearing constraining semantic information. Third, we hypothesized that prediction efficiency would correlate with autism symptom severity and autistic traits. Reduced prediction efficiency would be associated with higher levels of autism symptom severity and increased autistic traits.

## Methods

### Participants

We determined the sample size for this study through an a priori power analysis and by referencing relevant previous research. Eighteen participants per group would be required to detect a medium effect size (*f* = 0.25) with 95% statistical power at *α* = 0.05 (G*Power 3.1.9.7) [33]. However, to ensure robust findings, we established a more conservative recruitment target based on previous eye-tracking studies with similar designs [15, 16, 25], aiming to recruit no fewer than 40 participants per group. The final sample comprised 45 autistic children and 52 NT children matched for age, gender, and verbal IQ (see Table 1 for detailed participant characteristics). Post-hoc sensitivity analysis indicated that our final sample size provides 95% power to detect small-to-medium effects (*f* = 0.15) at α = 0.05.

**Table 1:** Participant demographics

	Autistic	NT	*p*
*N*	45	52	-
Gender (Male/Female)	40/5	45/7	0.767
Age range (years)	3.80–8.05	3.02–7.04	-
Age (years)	5.67 (0.98)	5.40 (0.92)	0.175
Verbal IQ	105.91 (17.15)	109.69 (10.93)	0.193
CARS-2	35.63 (3.03)	-	-
AQ-Child	89.45 (13.10)	48.62 (6.91)	< 0.001

NT = neurotypical. CARS-2 = Childhood Autism Rating Scale, Second Edition. AQ-Child = Autism Spectrum Quotient-Children’s Version. Standard deviations were in parentheses

Autistic children were recruited through professional support centers, whereas NT children were recruited online. The inclusion criteria for the autistic group required a diagnosis of autism spectrum disorder by a licensed pediatrician, as reported by parents. During the study, experienced psychologists administered the Childhood Autism Rating Scale, Second Edition (CARS-2) [34] to verify their diagnoses. Children were excluded if their parents reported comorbid physical or neurodevelopmental disorders (such as attention-deficit/hyperactivity disorder). For the NT group, parents completed the Autism Spectrum Quotient-Children’s Version (AQ-Child) [35]. Children were excluded from this group if they scored above the high-risk threshold for autism (76 out of 150 points) on the AQ-Child scale, or if parents reported any physical or neurodevelopmental disorders or related concerns. Parents of autistic children also completed the AQ-Child.

For participants under 7 years of age (42 in the autistic group, 51 in the NT group), the Mandarin Chinese version of the Wechsler Preschool and Primary Scale of Intelligence-Fourth Edition (WPPSI-IV) [36, 37] was administered to assess participants’ verbal IQ. For participants aged 7 years and above (3 in the autistic group, 1 in the NT group), the Mandarin Chinese version of the Wechsler Intelligence Scale for Children-Fourth Edition (WISC-IV) [38] was administered. For children under 4 years of age assessed with WPPSI-IV (1 in the autistic group, 4 in the NT group), verbal IQ was derived from the Information subtest (assessing general knowledge through picture selection and verbal responses) and the Receptive Vocabulary subtest (assessing receptive language through picture selection). For children aged 4 years and above assessed with WPPSI-IV (41 in the autistic group, 47 in the NT group), verbal IQ was derived from the Information subtest and the Similarities subtest (assessing verbal concept formation and reasoning ability) [36]. For children assessed with WISC-IV, verbal IQ was derived from the Similarities subtest and the Comprehension subtest (measuring verbal reasoning, comprehension and expression, and practical knowledge). These subtests evaluate children’s receptive and expressive vocabulary knowledge. All the participants demonstrated verbal IQs above 70, with no significant group difference (see Table 1).

This study was approved by Peking University’s research ethics committee (approval number: 2024-02-11). Written informed consent was obtained from the parents or legal guardians of all participants prior to their participation. All procedures were conducted in accordance with the Declaration of Helsinki.

### Stimuli

This study employs an eye-tracking task adapted from the visual world paradigm to measure children’s predictive language processing efficiency and examine their incremental processing strategies [39]. The visual world paradigm has been widely used to investigate children’s real-time language processing [17-19, 40, 41]. Drawing from Borovsky et al.‘s (2012) experimental design, we carefully controlled the semantic relations between objects in the visual arrays and sentence components to examine strategies of incremental language processing. In each trial, the children heard a simple SVO-structured sentence such as “*The bird eats worms*,” which contained two information cues (agent and action) that semantically matched the sentence-final object (patient). During sentence presentation, participants viewed a visual array containing four images, each corresponding to one of four conditions: a target patient (“worm”), an agent-related distractor (“nest” related to “bird”), an action-related distractor (“rice” related to “eat”), and an unrelated distractor (“building”). The agent-related distractor is semantically congruent with the agent but not the action, whereas the action-related distractor is semantically congruent with the action but not the agent. Only the target object simultaneously matched both the agent and the action. Upon hearing the action word, children should have acquired sufficient semantic information to generate predictive looks toward the target.

This design enables differentiation between potential incremental processing strategies [19]. If children adopted a staged elimination strategy consistent with the Cohort model [28], they would initially fixate on agent-related items and subsequently narrow focus to the target after hearing the verb. Conversely, if they utilize a strategy resembling the TRACE model [26, 29], they would distribute attention to both the target and action-related distractors after verb presentation, despite the action-related distractor being incompatible with the agent.

Following Hua et al.’s methodology [32], words for agents, actions, and patients were selected from the following sources to generate sentence stimuli: (a) word lists from standardized developmental assessment for toddlers and preschool-aged children—the MacArthur-Bates Communicative Development Inventories (MB-CDI) [42] and the Peabody Picture Vocabulary Test-Revised (PPVT-R) [43], and (b) vocabulary from popular picture book series for preschool-aged children (the Frog series by Max Velthuijs). The selected agents and patients were all concrete nouns that could be clearly depicted in images. Following Borovsky et al.’s methodology [19], sentence quadruplets were constructed by cross-pairing two agents and two verbs and then matching appropriate patients to create four simple SVO-structured sentences. For example, cross-pairing the agents (“bird” and “worker”) with the actions (“eat” and “build”) and matching appropriate patients yielded the following four sentences: (1) “The bird eats the worm,” (2) “The worker eats the rice,” (3) “The bird builds the nest,” and (4) “The worker builds the building.” The target object images corresponding to these four sentences were worm, rice, nest, and building, which were arranged equidistant from the center of the screen against a gray background to form a visual array.

For any sentence within the quadruplet, each image in the visual array corresponded to one of four conditions: target (i.e., the patient at the end of the sentence), agent-related distractor, action-related distractor, or unrelated distractor (unrelated to either agent or action). Within each sentence quadruplet, each word/image appeared once in each condition, thus creating a fully balanced design (see Table 2 for an example of sentence quadruplets). Consequently, each word/image could serve as its own control as its role changed across different sentences. This methodological approach effectively controlled for any inherent differences in the perceptual salience or attractiveness of individual words and objects. Using the method described above, 6 sentence quadruplets (24 sentences in total) were generated. All image stimuli were photographs with white backgrounds, depicting clear, unambiguous representations of their corresponding concepts, selected from standardized picture databases and online resources.

**Table 2:** Example of sentence quadruplet and corresponding image stimuli content

Sentence Quadruplet			Images Corresponding to the Four Conditions (Displayed Simultaneously)			
Agent	Action	Patient	Target	Agent-related Distractor	Action-related Distractor	Unrelated Distractor
“The bird	eats	the worm.”	worm	nest	rice	building
“The worker	eats	the rice.”	rice	building	worm	nest
“The bird	builds	the nest.”	nest	worm	building	rice
“The worker	builds	the building.”	building	rice	nest	worm

Counterbalancing procedures were implemented to control for position and order effects. Within each sentence quadruplet, each condition appeared once in each of the four screen positions, ensuring equal presentation frequency across all locations. Two pseudo-randomized trial sequences were generated with constraints that (1) target images could not appear in the same position for more than two consecutive trials, and (2) sentences from the same quadruplet were never presented consecutively. In the formal experiment, participants were randomly assigned to one of these sequences.

Prior to the formal experiment, following Borovsky et al. [19], two preliminary validation experiments were conducted to evaluate the familiarity of stimulus materials to children. Fifteen NT children who did not participate in the formal experiment (including 7 females; *M* = 5.64 years, *SD* = 1.13 years, range = 3.48–7.11 years) were recruited for these two preliminary experiments. In the first experiment, children were asked to identify the four target images in each image sequence. All children correctly identified all the target images, confirming that the target words and their corresponding images selected for this experiment were familiar and recognizable to children. In the second experiment, children were asked to judge which image in the sequence could serve as the target image when only the agent and action were provided (e.g., “What would a cat catch?” or “What would a frog jump onto?“). The accuracy of judgment for all sentences exceeded 85% (*M* = 97.2%, *SD* = 16.5%), indicating that when provided with agent and action information, children possessed the necessary semantic knowledge to select the correct patient target. The complete list of sentence stimuli is provided in Table S1 in Supplemental Materials.

The audios of the sentences were generated via a text-to-speech artificial intelligence tool [44] featuring a young adult female voice speaking Mandarin Chinese with a sampling rate of 48 kHz. Adobe Audition software (2024 version) was used to edit each audio stimulus, removing the silent portion at the beginning of sentences and standardizing the onset times of different sentence components across all stimuli: action words commenced 750 ms after sentence onset, patient words commenced 1300 ms after sentence onset, and all sentence stimuli lasted for 2100 ms. This standardization of onset times ensured that children had equivalent temporal windows to utilize agent and action information to anticipate the target word across all sentences. All audio stimuli were evaluated by a Chinese language expert specializing in child language to ensure that the audio editing did not make the stimuli sound unnatural. All image stimuli were also evaluated by the same expert to confirm their suitability for children.

### Procedure

Participants were seated at approximately 65 cm from a 24-inch computer screen (resolution: 1920 × 1080), with head movement unconstrained to maintain natural viewing conditions appropriate for young children. A Tobii Pro X3-120 eye tracker [45] was used to record children’s eye movements. The eye tracker operates at a sampling rate of 120 Hz with a gaze accuracy of 0.4° and gaze precision of 0.24° under binocular measurement conditions [45]. Prior to the experimental task, all participants completed a standard five-point calibration procedure. Calibration was considered successful when the average accuracy across all five calibration points was better than 1.5° of visual angle, with no individual point exceeding 2.0° [46]. Calibration was repeated until a satisfactory calibration was obtained.

**Fig. 2:**
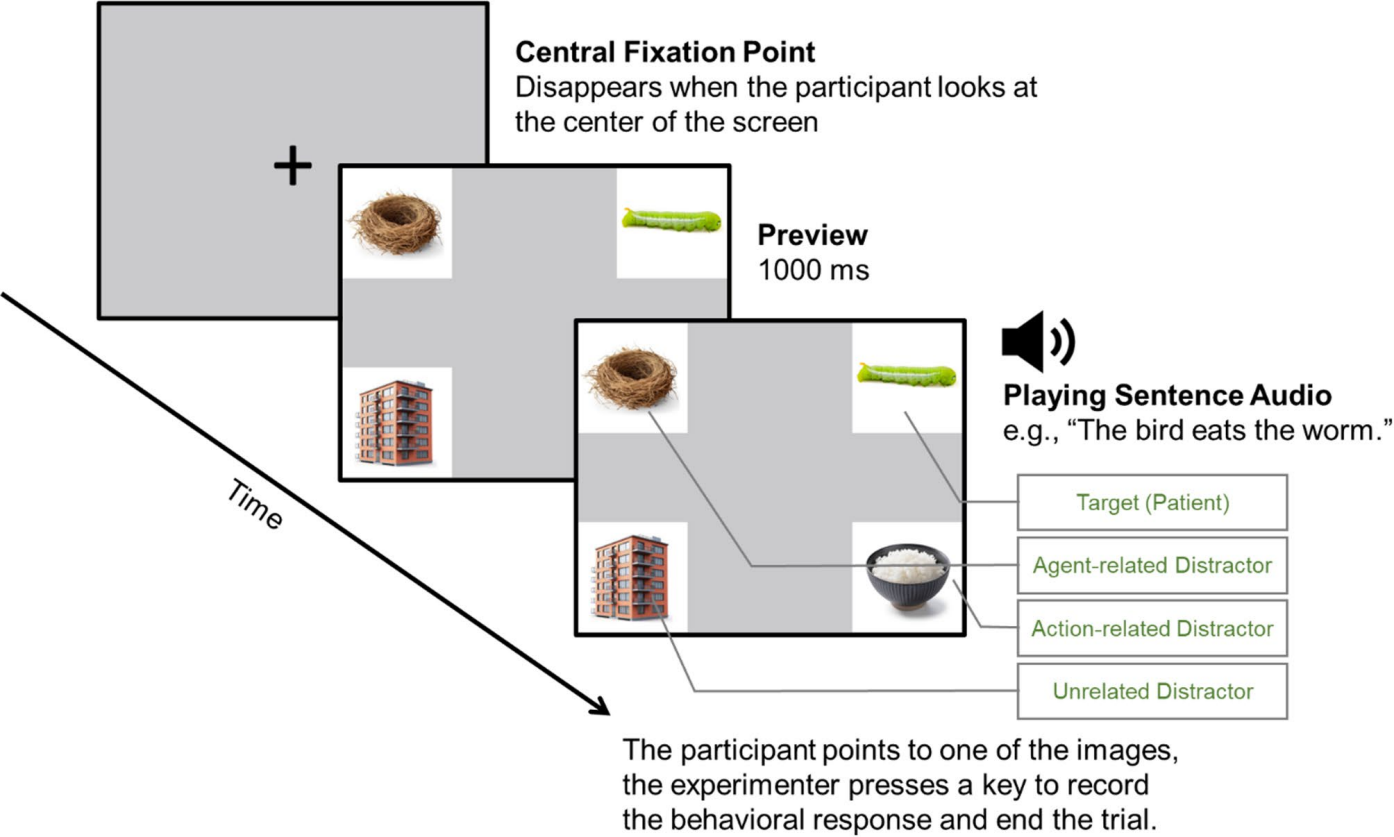
Visual world paradigm sample trial

The task comprised 2 practice trials and 24 formal trials, taking approximately 5–10 min in total. Each trial began with a central fixation point on the screen. Once the eye tracker detected the participant’s fixation at this point, four object images were displayed on the screen, arranged equidistant from the center (see Fig. 2 for a sample trial). Each image corresponded to one of four conditions: target patient, agent-related distractor, action-related distractor, or unrelated distractor. After a 1000-ms preview period, the sentence audio was played through loudspeakers. Children were instructed to use a pointer to indicate the image that “matched the sentence,” after which the experimenter pressed a key to record the response and end the trial. Compared to traditional response methods such as mouse clicking or directly touching the screen, this method helped maintain an optimal and stable distance between the participant and the eye tracker, avoided screen contamination from hand contact, and ensured young participants’ engagement with the task.

### Data analysis

Eye-tracking data preprocessing was conducted using MATLAB R2023b [47], and all statistical analyses were performed using R version 4.2.3 [48]. Eye movement data were preprocessed using the Tobii I-VT fixation filter [49]. Missing data due to eye blinks were interpolated linearly with a maximum gap of 75 ms. Gaze positions from both eyes were averaged to calculate fixations, or monocular data were used when binocular data were unavailable. Adjacent fixations were merged with a maximum time interval of 75 ms and maximum angle of 0.5°, and fixations shorter than 100 ms were excluded from analysis [50, 51]. We defined areas of interest (AOIs) for the four conditions as square regions corresponding to the stimulus pictures, with equivalent sizes across all conditions (384 × 384 pixels, approximately 10.6 cm × 10.6 cm on the 24-inch screen with 1920 × 1080 resolution). The analysis window spanned from sentence onset to 400 ms after the sentence offset (i.e., 0 to 2500 ms, as each sentence audio lasted 2100 ms). The endpoint was set at 400 ms after sentence offset to capture delayed effects due to the relatively brief sentence duration [52]. To examine fine-grained temporal patterns of fixation behavior, the analysis window was segmented into 50-ms time bins, with fixations binary-coded based on whether the corresponding AOI received fixation within each time bin [52, 53]. The binary-coded fixation data were subsequently averaged across trials and participants to obtain time-course estimates of fixation proportions.

**Fig. 3:**
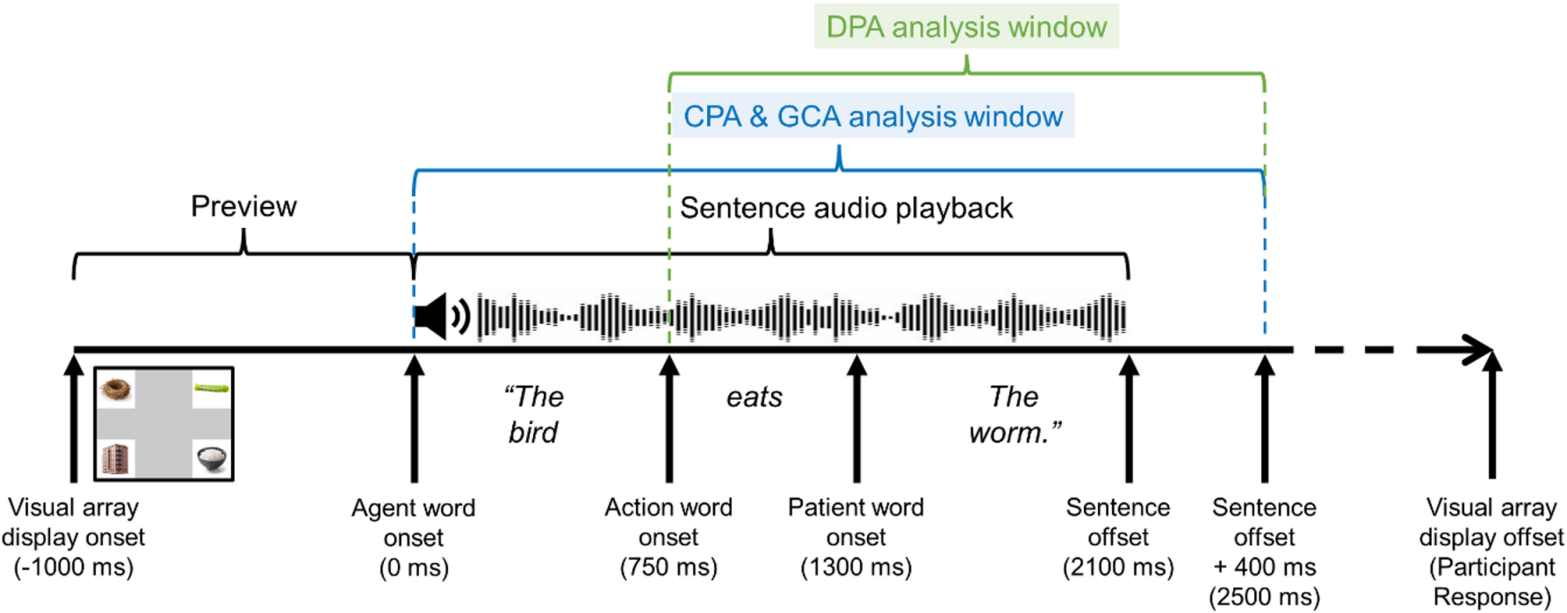
Trial timeline and analysis windows. DPA = divergence point analysis. CPA = cluster-based permutation analysis; GCA = growth curve analysis

We employed three complementary analytical approaches to examine the temporal dynamics of predictive fixations in autistic and NT groups: cluster-based permutation analysis (CPA) [54], growth curve analysis (GCA) [55] and divergence point analysis (DPA) [56]. CPA was utilized to identify sustained periods where fixations of one condition significantly differed from those of another condition in the entire analysis window (i.e., 0-2500 ms). This approach is particularly useful for identifying when participants consistently demonstrate biased looking toward specific objects during sentence processing. GCA was used to compare the two groups’ fixation trajectories to the target throughout the entire analysis window (i.e., 0-2500 ms). This method enables modeling of continuous changes in fixation proportions over time, accounting for both linear and nonlinear trends, and identifying potential group differences in these trajectories. DPA was employed to determine the time point at which predictive fixation behavior emerged for each group and to assess whether significant group differences existed in the timing of predictive fixation behavior. The analysis window for DPA spanned from verb onset to 400 ms after sentence offset, thereby identifying the time point at which participants redirected their attention from the agent-related distractor to the target after hearing the verb (see Fig. 3 for analysis windows of each analytical method). Together, these three analytical approaches provide a comprehensive perspective on different aspects of predictive language processing: the CPA determines specific temporal windows where sustained biased attention to certain objects occurs, the GCA reveals general patterns and group differences in overall fixation trajectories, and the DPA identifies the precise timing of critical predictive shifts in attention. This multimethod approach allows us to examine both global patterns and specific time-course features of predictive language processing in the autistic and NT groups.

## Results

We calculated the proportion of trials where participants correctly identified the target image. Accuracy was very high for both the autistic group (*M* = 93.4%, *SD* = 10.5%) and the NT group (*M* = 95.9%, *SD* = 7.3%). Generalized linear mixed model analysis showed no significant group difference in behavioral accuracy (*B* = 0.39, *SE* = 0.83, *p* =.640).

### Fixation patterns and incremental processing strategy

**Fig. 4:**
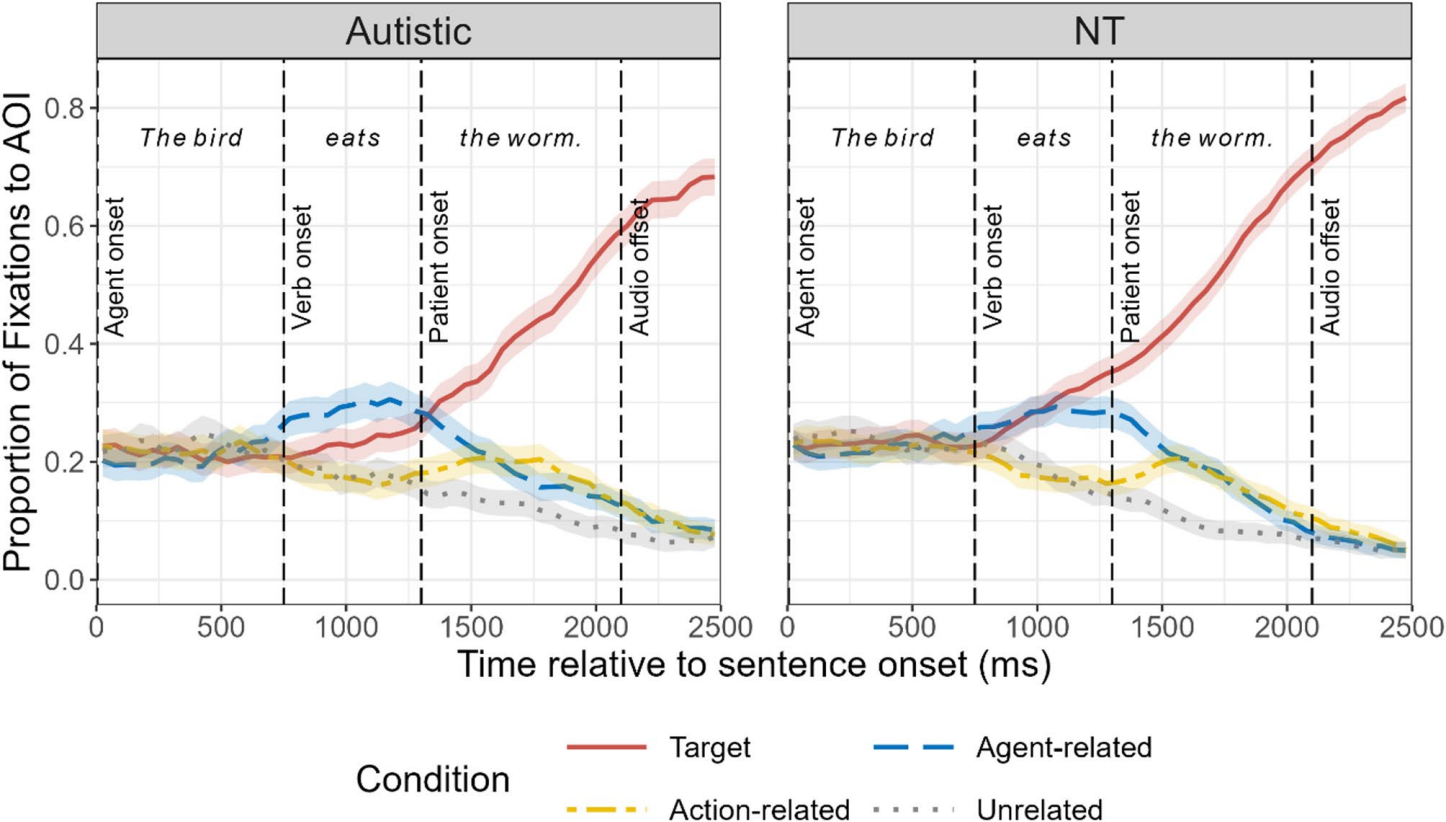
Time course of fixations to the target compared to the agent-related, action-related, and unrelated distractors. AOI = area of interest. NT = neurotypical. Ribbons represent 95% confidence intervals

We calculated and plotted the mean fixation proportions for each AOI in 50-ms bins from sentence onset to 400 ms after sentence offset for the autistic and NT groups (see Fig. 4). Both groups exhibited similar overall fixation patterns: Upon hearing the agent, fixations shifted toward both the target and agent-related distractor, demonstrating agent-based prediction; subsequently, after hearing the verb, fixations systematically shifted toward the target patient (relative to all distractors), demonstrating verb-based prediction. Following this shift, fixations predominantly remained on the target for the remainder of the sentence duration. Notably, shortly after verb onset, both groups also demonstrated an increase in fixations to the action-related distractor, despite its incompatibility with the agent.

**Fig. 5:**
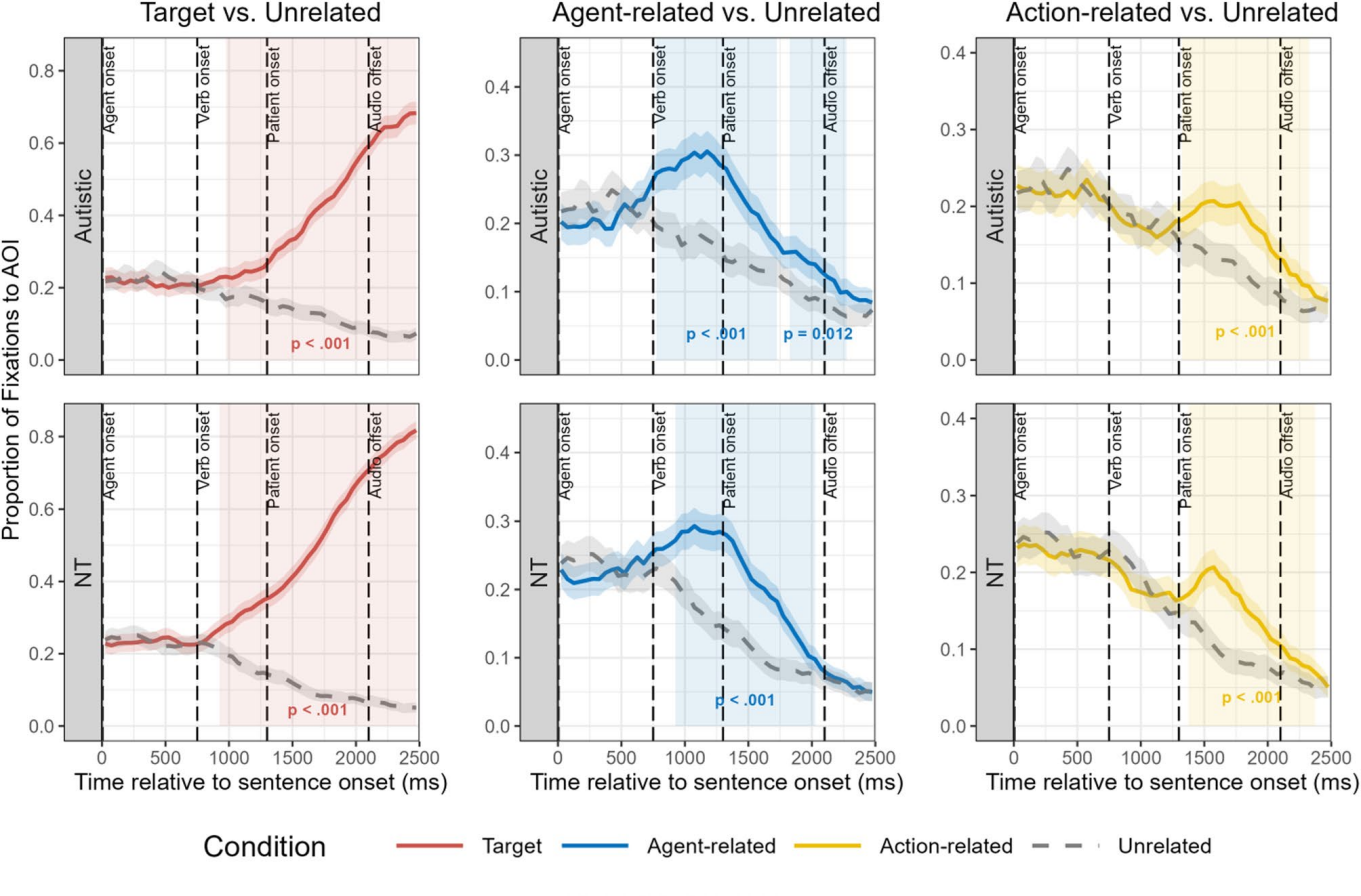
Cluster-based permutation analyses results. The figure shows the proportion of fixations over time to different AOIs compared to the unrelated distractor, with significant clusters identified through cluster-based permutation analyses. Ribbons represent 95% confidence intervals. Semi-transparent colored areas indicate statistically significant time clusters identified by CPA (*α* = 0.05, 1000 permutations), where fixations to the critical AOI significantly differed from the unrelated AOI. The *p*-values for each significant cluster are displayed within each panel

To confirm the qualitative characteristics of the fixation patterns we observed, we further conducted CPAs [54] to quantitatively identify periods of sustained biased looking toward the target, agent-related distractor, and action-related distractor against the unrelated distractor (see Fig. 5). We used 1000 random permutations with a cluster-forming threshold of *α* = 0.05. Temporal adjacency was defined as contiguous 50-ms time bins, with clusters formed when two or more adjacent time points showed effects in the same direction. The sum of the *t*-statistics within each cluster (Σ*t*) served as the cluster statistic, with clusters considered significant at *p* <.05 when compared against the empirical null distribution.

For the target versus unrelated comparison, the CPA revealed significant clusters for both groups: the autistic group demonstrated significant biased looking toward the target from 975 to 2475 ms (Σ*t* = 333.12, *p* <.001), while the NT group showed a similar pattern from 925 to 2475 ms (Σ*t* = 545.90, *p* <.001). These results indicate that both groups demonstrated robust biased looking toward the target shortly after verb onset that persisted throughout the remainder of the trial; however, the magnitude of the effect was stronger in NT children, as shown by the higher cumulative *t*-statistic in the NT group (Σ*t* = 545.90 vs. 333.12). For the agent-related vs. unrelated comparison, the autistic group presented two significant clusters: one from 775 to 1725 ms (Σ*t* = 89.95, *p* <.001) and another from 1825 to 2275 ms (Σ*t* = 27.77, *p* =.012). The NT group showed a significant cluster from 925 to 2025 ms (Σ*t* = 132.61, *p* <.001). These results indicate that both groups demonstrated biased looking toward the agent-related distractor during sentence processing, reflecting agent-based prediction. Similarly, both groups also showed significant clusters in the action-related vs. unrelated comparison: the autistic group from 1325 to 2325 ms (Σ*t* = 77.98, *p* <.001) and the NT group from 1375 to 2375 ms (Σ*t* = 83.66, *p* <.001). The emergence of these significant biased looking toward action-related distractors shortly after verb onset provides quantitative evidence that both groups employed a TRACE-like incremental processing strategy [26, 29]. Rather than using a staged elimination approach that would only maintain fixations to items compatible with both agent and verb information, both groups increased fixations to items related to the verb even though they were incompatible with the agent. Thus, both autistic and NT children maintain activation of multiple semantically related candidates during language processing. The timing of these significant clusters further reveals similarities in the overall processing sequence across groups, with agent-based effects emerging first, followed by parallel activation of both target and action-related fixations after verb onset.

### Group difference in overall trajectories of predictive fixations

We examined the group difference in the overall trajectory of predictive fixations by conducting a GCA [55]. Instead of directly comparing fixation proportions to the target between groups, we used the extent to which participants demonstrate biased looking to the target compared to the unrelated distractor to reflect predictive fixations, so as to better isolate effects related to predictive processing while controlling for potential group differences in overall visual attention levels [57]. Specifically, we computed the log-gaze proportion ratio between target and unrelated distractor for each 50 ms time bin using the following formula [32, 52, 58, 59]:

$$\begin{array}{l}\\\:\text{l}\text{o}\text{g}\text{-}\text{g}\text{a}\text{z}\text{e}\:\text{p}\text{r}\text{o}\text{p}\text{o}\text{r}\text{t}\text{i}\text{o}\text{n}\:\text{r}\text{a}\text{t}\text{i}\text{o}=\\\text{log}\left(\frac{\text{fixation\:on\:target}+0.5}{\text{fixation\:on\:unrelated\:distractor}+0.5}\right)\end{array}$$

The log-gaze proportion ratio offers a standardized measure of target-to-unrelated distractor fixation bias and is particularly suitable for our task design where the target and distractors are simultaneously present [52]. The log-gaze proportion ratio was expected to be around zero (indicating no biased looking) initially and increase as children generated predictions and oriented attention to the target overtime.

**Fig. 6:**
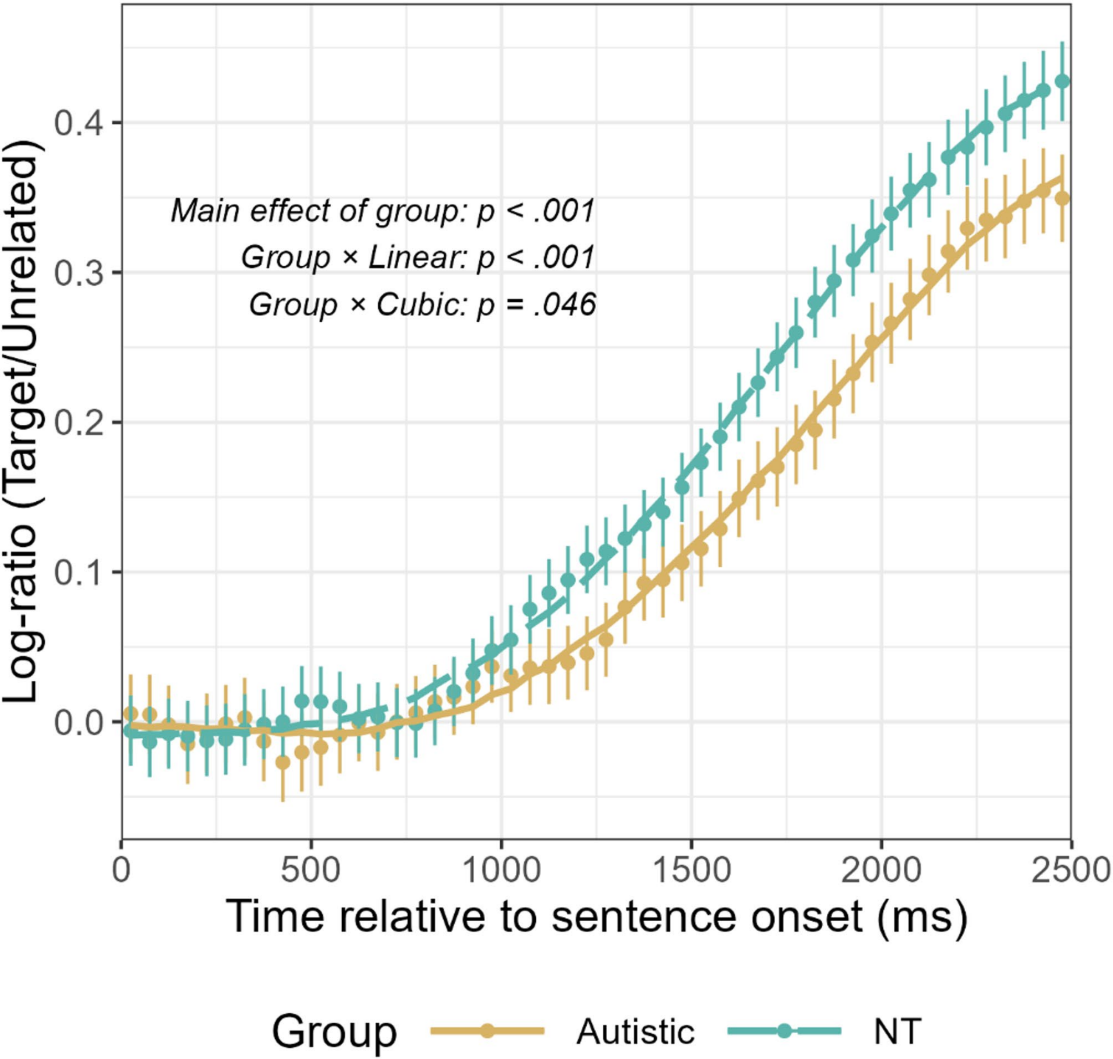
Model fit for growth curve analysis for log-gaze proportion ratios. NT = neurotypical. Error bars represent 95% confidence intervals. A fourth-order (quartic) orthogonal polynomial model was adopted. As shown in the plot, the autistic group exhibited an overall reduced log-ratio and a lower rate of increase in the log-ratio than the NT group

To determine the optimal model for our data, we conducted a thorough model selection process (see Supplemental Materials S2 for detailed description). The final model fitted to the log-ratio data was a linear mixed-effects model with fixed effects including orthogonal time polynomials (up to quintic) and their interactions with group. Random intercepts were included for participants and trials. The NT group was set as the reference level. GCA model statistics and model fit visualization are presented in Table 3; Fig. 6, respectively.

**Table 3:** GCA results of target fixation proportion time course

Fixed Effects	*B*	*SE*	*t*	*p*
(Intercept)	0.13	0.01	9.34	**< 0.001**
Linear	0.95	0.01	104.96	**< 0.001**
Quadratic	0.28	0.01	30.90	**< 0.001**
Cubic	-0.07	0.01	-7.95	**< 0.001**
Quartic	-0.05	0.01	-5.35	**< 0.001**
Group	-0.02	0.01	-3.57	**< 0.001**
Linear × Group	-0.10	0.01	-11.25	**< 0.001**
Quadratic × Group	0.01	0.01	1.28	0.199
Cubic × Group	0.02	0.01	2.00	**0.046**
Quartic × Group	0.004	0.01	0.47	0.640

GCA = growth curve analysis. *B* = unstandardized coefficient. *SE* = standard error. Linear, Quadratic, Cubic, Quartic, and Quintic refer to orthogonal polynomial time terms. NT children served as the reference group. Significant *p*-values are shown in bold

Results revealed significant effects for all time polynomials: linear (β = 0.95, *SE* = 0.01, *p* <.001), quadratic (β = 0.28, *SE* = 0.01, *p* <.001), cubic (β = -0.07, *SE* = 0.01, *p* <.001), and quartic (β = -0.05, *SE* = 0.01, *p* <.001). These effects capture the complex, nonlinear trajectory of log-ratio over time across all participants. Analysis further revealed a significant main effect of group (β = -0.02, *SE* = 0.01, *p* <.001), indicating that the overall log-ratio for the autistic group was significantly lower than that of the NT group. This suggests that autistic children, relative to NT children, showed overall reduced biased looking to the target compared to the unrelated distractor. The interaction between group and the linear time term was also significant (β = -0.10, *SE* = 0.01, *p* <.001), demonstrating that the increase in the log-ratio was significantly slower for the autistic group compared to the NT group. Additionally, there was a significant interaction between group and the cubic time term (β = 0.02, *SE* = 0.01, *p* =.046), reflecting subtle differences in the inflection patterns of the trajectories of log-ratio between groups. Interactions between group and the quadratic (β = 0.01, *SE* = 0.01, *p* =.199) and quartic (β = 0.004, *SE* = 0.01, *p* =.640) terms were not significant.

To ensure that these group differences were not attributable to the group difference in verbal IQ distributions, given the broader range of verbal IQ scores in the autistic group (77–146) compared to the NT group (83–126), we conducted an additional analysis including verbal IQ as a covariate (see Supplemental Materials S3 for detailed results). The critical group effects remained significant after controlling for verbal IQ, confirming that the observed differences in predictive fixation trajectories reflect autism-specific mechanisms.

These results demonstrate that while both autistic and NT groups oriented their attention to the target relative to the unrelated distractor over time, demonstrating predictive fixations as sentences unfolded, the autistic group exhibited reduced biased looking to the target overall and slower rates of growth in their biased looking to the target compared to the NT group. Importantly, because our analysis controlled for potential group differences in general visual attention by using log-gaze proportion ratios, these results suggest reduced prediction efficiency during language comprehension in autistic children.

### Group difference in onset of predictive looking

**Fig. 7:**
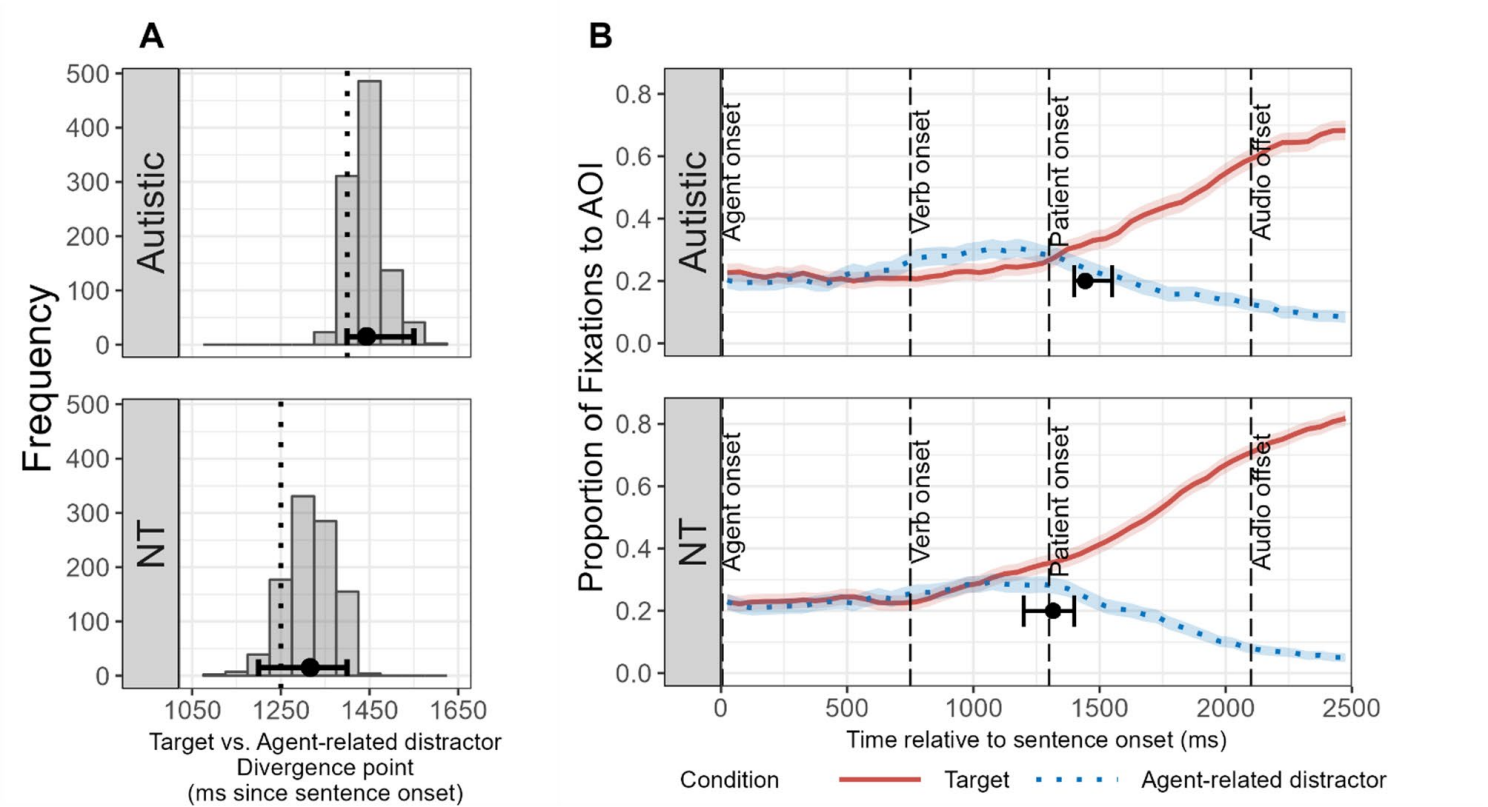
Divergence point analyses results. AOI = area of interest. NT = neurotypical. **A** Bootstrap distributions of divergence points (number of iterations *N* = 1000) in fixation curves between target and agent-related distractor for each group. Black dots and their error bars represent the bootstrap estimates of divergence points and 95% confidence intervals. Dotted vertical lines indicate the divergence points in the original data. **B** Divergence points and 95% confidence intervals superimposed on the fixation curves for Target versus Agent-related distractor

We then examined the temporal onset of predictive looking to the target for each group. Specifically, we used DPA [56] to determine the time point at which each group began to demonstrate biased looking toward the target compared to the agent-related distractor. Given that participants needed to hear the verb to predict the patient of the spoken sentence and thus redirect their attention from the agent-related distractor toward the target, our analysis focused on the time window from verb onset to 400 ms after sentence offset (i.e., from 750 ms to 2500 ms). Bootstrap analysis (1000 iterations) revealed that the estimated divergence point for the autistic group occurred at 1443.4 ms after sentence onset (95% confidence interval [1400, 1550]), whereas for the NT group it occurred at 1316.2 ms (95% CI [1200, 1400]). Given that the sentence-final patient word began at 1300 ms after sentence onset, and accounting for the estimated time (approximately 200 ms) required to generate an eye movement based on phonological information [17, 60, 61], these divergence points indicate that both groups demonstrated predictive fixations to the target before processing the sentence-final patient word. That is, both groups were able to utilize semantic information in the sentence to predict upcoming information. The distributions of the estimated divergence points and their overlays on the fixation curves are shown in Fig. 7.

To examine the group difference in the estimated divergence points, we conducted an additional bootstrap analysis with 1000 iterations. The results showed that the NT group reached the divergence point significantly earlier than did the autistic group. There was a difference of 127.2 ms between the two groups (95% CI [-250, 0]), and the difference in divergence points was significant (*p* =.013). This finding indicates that after hearing the verb, the NT group demonstrated biased looking toward the target compared to the agent-related distractor more rapidly than the autistic group. The DPA results further suggest that compared to the autistic group, the NT group oriented their attention to the target more rapidly, indicating faster predictive processing.

### Associations between prediction efficiency and autism symptomatology/autistic traits

**Fig. 8:**
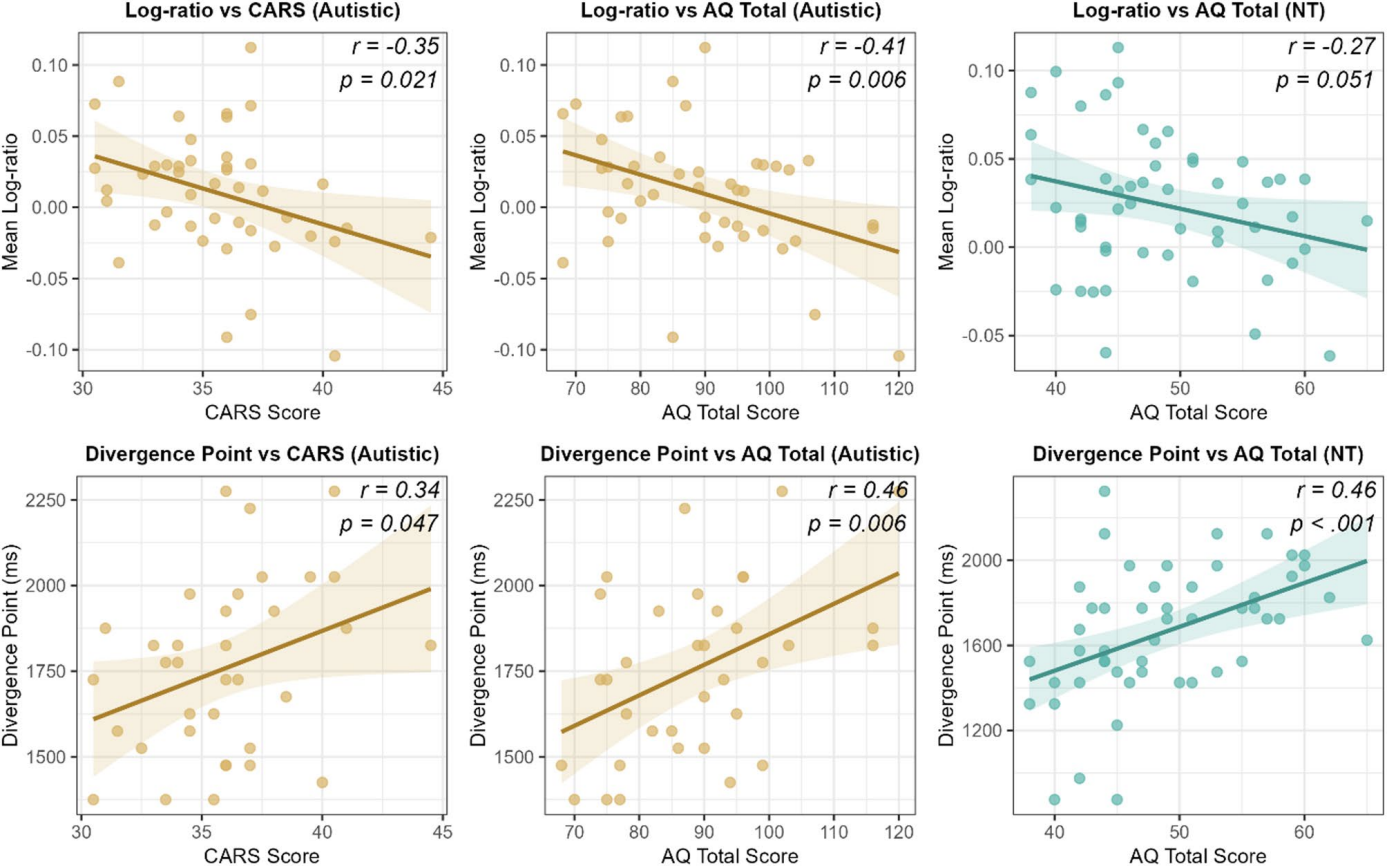
Correlations between prediction efficiency indices and autism-related measures. Each panel includes a fitted regression line with 95% confidence interval. Correlation coefficients and *p*-values are displayed in each panel

Given that our group-level analyses revealed significant differences in prediction efficiency between autistic and NT children, we next examined whether prediction efficiency varied with autism symptom severity and autistic traits. Specifically, we first computed two prediction efficiency indices for each participant: (1) individual log-ratio scores were calculated by averaging log-gaze proportion ratios between target and unrelated distractor across the prediction time window (from agent onset to patient onset, i.e., 0-1300 ms), providing an index of overall predictive looking bias; (2) individual divergence points were calculated as the time at which participants began to show sustained predictive looking toward targets compared to agent-related distractors, providing a measure of prediction onset timing. Then we conducted Pearson correlation analyses between prediction efficiency indices and autism-related measures: autism symptom severity in the autistic group (measured by CARS) and autistic traits across both groups (measured by AQ). Figure 8 presents the relations between prediction efficiency indices and autism-related measures.

In the autistic group, CARS scores showed significant negative correlations with log-ratio scores (*r* = −.35, *p* =.021, 95% CI [-0.59, − 0.06]), indicating that higher autism symptom severity was associated with reduced prediction efficiency. Similarly, AQ total scores were significantly negatively correlated with log-ratio scores in the autistic group (*r* = −.41, *p* =.006, 95% CI [-0.63, − 0.13]). In the NT group, the correlation between AQ total scores and log-ratio scores was negative and marginally significant (*r* = −.27, *p* =.051, 95% CI [-0.51, 0.00]). For divergence points, CARS scores in the autistic group showed a significant positive correlation (*r* =.34, *p* =.047, 95% CI [0.01, 0.60]), indicating that higher symptom severity was associated with delayed onset of predictive looking. AQ total scores were positively correlated with divergence points in both the autistic group (*r* =.46, *p* =.006, 95% CI [0.14, 0.68]) and the NT group (*r* =.46, *p* <.001, 95% CI [0.20, 0.65]), suggesting that increased autistic traits were consistently associated with slower prediction onset across groups.

Exploratory analyses examining associations between prediction efficiency and specific AQ dimensions revealed that the communication subscale—which measures autism-related communication difficulties such as challenges in engaging in reciprocal communication, understanding conversational cues, and interpreting social language nuances, with higher scores indicating greater difficulties [35]—showed the most consistent associations with prediction efficiency across groups. After FDR correction for multiple comparisons across the five AQ subscales, communication scores remained significantly correlated with both prediction efficiency indices across groups. Specifically, communication scores showed negative correlations with log-ratio scores in both the autistic group (*r* = −.45, FDR-*p* =.012) and NT group (*r* = −.41, FDR-*p* =.012), and positive correlations with divergence points in both the autistic group (*r* =.49, FDR-*p* =.009) and NT group (*r* =.51, FDR-*p* <.001), indicating that reduced prediction efficiency was consistently associated with increased autism-related communication difficulties across diagnostic groups. Additionally, in the autistic group, both social skills scores (*r* = −.34, FDR-*p* =.040) and attention switching scores (*r* = −.35, FDR-*p* =.040) showed significant correlations with log-ratio scores, while attention switching scores also correlated significantly with divergence points (*r* =.48, FDR-*p* =.009). In the NT group, social skills scores were significantly associated with divergence points (*r* =.38, FDR-*p* =.010). These results indicate that difficulties in social skills and attention switching were also associated with reduced prediction efficiency. See Supplemental Materials S4 for detailed correlation analysis results for AQ dimensions.

## Discussion

This study aimed to investigate incremental processing strategy and prediction efficiency during spoken language comprehension in autistic children compared to NT children. Using the visual world paradigm, we compared eye movement patterns of autistic children (3 to 8 years old) and age-, gender- and verbal-IQ matched NT children during comprehension of simple SVO-structured sentences. The study employed visual arrays containing target objects and three types of distractors (agent-related, action-related, and unrelated) to assess how children utilize and integrate semantic cues in sentences to predict upcoming information. The main research findings are: (1) Both autistic children and NT children were able to utilize and integrate semantic cues in sentences to predict upcoming linguistic content, with both groups adopting an incremental processing strategy consistent with the TRACE model [26, 29] rather than a staged elimination approach [28] (Figs. 4 and 5); (2) Autistic children demonstrated reduced prediction efficiency compared to NT children, as manifested by lower overall fixation proportions to and slower growth rates of fixation proportions to target objects relative to unrelated distractors (Fig. 6), and delayed onset of predictive fixations to target objects relative to NT children (Fig. 7); (3) Reduced prediction efficiency was associated with higher levels of autism symptom severity in the autistic group and increased autistic traits across groups (Fig. 8), with autism-related communication difficulties showing the most robust associations across groups.

The first main finding of this study is that both autistic and NT children were able to utilize semantic information in sentences to predict upcoming information. Both groups exhibited predictive fixations to target objects before hearing the target word (Figs. 4 and 5), supporting the view in existing literature that autistic children retain linguistic prediction abilities [11, 15, 17, 18]. Furthermore, both autistic and NT children employed a TRACE-like incremental processing strategy [26], as was observed in previous studies with NT children and adults [19, 30, 40]. In our task, shortly after hearing the verb, both groups not only redirected fixations toward the target object but also increased fixations to the distractor that was semantically related to the verb, despite its incompatibility with the agent (Figs. 4 and 5). This fixation pattern suggests that autistic children, similar to NT children, flexibly integrate multiple aspects of semantic information and maintain the activation of multiple candidates during language comprehension. This finding indicates fundamental similarities in semantic processing strategies between autistic and NT children, complementing previous research on other aspects of semantic processing in autism that also identified comparable patterns between groups [16, 31, 32]. This incremental processing strategy allows autistic children to more flexibly adapt to changes and unexpected elements in linguistic input during language comprehension [19]. Since less probable options are not completely eliminated, even when expressive or receptive errors occur in the initial stage, they can more efficiently adjust predictions through subsequent cues [19]. Such flexibility is particularly important for communication in naturalistic contexts, where linguistic input is often ambiguous and variable.

Notably, although autistic children demonstrated a similar incremental processing strategy to that of age- and language ability-matched NT children, they showed reduced prediction efficiency compared with that of NT children during spoken language comprehension. Specifically, autistic children showed significantly lower overall fixation proportions and fixation proportion growth rates to target objects relative to unrelated distractor than NT peers (Fig. 6). This finding aligns with Zhou et al. [18], who found that 5-year-old autistic children demonstrated reduced fixation proportions to target objects during predictive language processing compared to age-matched NT children. Similarly, Bavin et al. [15] found that autistic children aged 5 to 9 years shifted their fixations away from target objects more quickly after predictive fixations compared to age-matched NT children. Zhou et al. [18] and Bavin, Kidd et al. [15] proposed that disparities in fixation patterns to target objects might reflect differences in cognitive control abilities for visual attention between the two groups. Such explanation was supported by Huettig et al.’s [57] finding that autistic children’s linguistic prediction was associated with their non-predictive visual attention behaviors, suggesting that differences in predictive eye movement patterns might at least partially derive from underlying differences in visual attention. Future investigations should further explore the potential associations between performance differences in predictive language processing and nonlinguistic cognitive abilities, particularly attentional control mechanisms and visual processing tendencies in autism.

Moreover, autistic children showed delayed onset timing of predictive looking toward target objects (Fig. 7). This finding corroborates results from Bavin, Kidd et al. [15] and Bavin, Prendergast et al. [25], who observed that autistic children responded more slowly when integrating constraining information (adjectives, nouns) in sentences to predict targets. The reduced prediction efficiency in autistic children observed in our study align with several prediction-based theoretical accounts of autism, though these accounts propose different underlying mechanisms. For example, the Bayesian accounts of autism proposed by Pellicano and Burr suggests that autistic individuals may have attenuated or less precise priors, leading to perception that is less influenced by top-down predictions [22, 62, 63]. Under this framework, the reduced prediction efficiency in autistic children might reflect their difficulties in the formation and utilization of prior knowledge representations based on recent linguistic context. In contrast, the HIPPEA (high, inflexible precision of prediction errors in autism) account by Van de Cruys et al. proposed that rather than imprecise predictions, autistic individuals actually form precise expectations but assign inflexibly high weight to prediction errors, making them particularly sensitive to violations of their predictions [23]. According to this account, the delayed predictive looking we observed might result not from imprecise predictions, but from difficulties in flexibly updating predictions when initial semantic cues (like agent information) need to be integrated with subsequent information (like verb constraints). The PIA (predictive impairment in autism) hypothesis proposed by Sinha et al. [3] focuses on a more fundamental deficit: inaccuracies in estimating conditional probabilities between events. According to PIA, autistic individuals have an altered “association sensitivity function” that makes it harder to detect predictive relationships. In the context of our findings, the internally established probabilistic relationships between stimulus objects or linguistic elements (e.g., a conditional probability that edible objects follow eating verbs) may be weaker for autistic children, resulting in reduced prediction efficiency. Overall, the reduced prediction efficiency we observed provides empirical support for prediction-based theories of autism, though future research will be needed to distinguish between these mechanistic accounts and determine which aspects of predictive processing are most affected in autism.

The correlation analyses provide further insights into the potential role of prediction difficulties in autism. Reduced prediction efficiency was associated with higher levels of autism symptom severity in the autistic group and increased autistic traits across groups (Fig. 8), supporting theoretical perspectives that position prediction difficulties as a fundamental characteristic of autism [3, 22, 23]. Furthermore, regarding dimensions of autistic traits, reduced prediction efficiency and autism-related communication difficulties showed the strongest and most consistent associations. This pattern aligns with Huettig et al. [57]’s finding that autistic children exhibiting reduced predictive looking scored lower in standardized communication assessments, though our findings extend this by specifically examining autism-related communication patterns measured by AQ (e.g., challenges in engaging in reciprocal communication, understanding conversational cues, and interpreting social language nuances) [35]. The strong correlations between reduced prediction efficiency and these communication difficulties may reflect the predictive processing demands inherent in social communication. Successful communication requires rapid integration of multiple semantic cues and efficient prediction of context-dependent linguistic information [4]. Additionally, social communication involves multi-modal processing, integrating both verbal and non-verbal cues such as facial expressions, gestures, and prosody [64], areas where autistic individuals commonly experience difficulties in both understanding and production [1, 65]. When prediction efficiency is reduced, autistic children may struggle to keep pace with the dynamic, fast-flowing nature of natural conversations, which could contribute to the broader communication challenges observed in autism.

Beyond communication, reduced prediction efficiency also showed associations with social skills and attention switching difficulties in the autistic group. Social skills difficulties, conceptualized as reduced confidence and ease in social situations and less preferences for social activities and comfort with social interaction in the AQ scale, may stem partly from predictive processing challenges, as successful social interaction requires rapid prediction and adjustment based on dynamic social cues [3, 22]. Attention switching difficulties, manifested as challenges in switching focus between tasks or activities and adapting to changes in routine or unexpected events, may also be partly attributable to reduced prediction efficiency, as less efficient predictive mechanisms could make environmental changes and unpredictable situations more overwhelming and difficult to navigate [23].

Beyond providing potential explanations for the autism-related difficulties discussed above, prediction impairments may also account for distinctive language and behavioral patterns characteristic of autism, which could represent compensatory strategies developed in response to predictive processing challenges—an avenue warranting further empirical investigation: For instance, echolalia (repetitive speech) [66, 67] may serve as a compensatory strategy when predictions about upcoming linguistic content are formed more slowly, reducing the need to generate novel, contextually appropriate responses. Similarly, restricted and repetitive patterns of behavior may represent another compensatory strategy for navigating an environment that appears unpredictable due to reduced prediction efficiency [3, 22, 23]. When linguistic prediction requires more time and cognitive resources, autistic children may rely on familiar, repetitive verbal routines and conversational scripts—such as reciting memorized content, using stereotyped phrases, or insisting on specific verbal exchanges [66, 68]—to create a more manageable linguistic environment. From a practical perspective, interventions targeting language development of autistic children might benefit from considering the reduced prediction efficiency we observed, which might involve providing additional processing time during language activities, using highly constraining semantic contexts to facilitate prediction, and gradually increasing the semantic integration demands for prediction. Future research should further investigate associations between prediction during language comprehension and core features of autism to better understand their underlying mechanisms and inform targeted interventions.

Our study makes several unique contributions to the existing literature. To our knowledge, this is the first study to examine incremental processing strategies during real-time sentence comprehension in autistic children, moving beyond single-cue prediction tasks to investigate how they integrate multiple semantic elements (agent and action). Our comprehensive methodological approach combining CPA, GCA, and DPA captures different temporal aspects of predictive processing that single analytical methods cannot provide. Additionally, by adopting both group-level and dimensional perspectives, we provide empirical support for prediction-based theories of autism while offering insights into potential mechanisms by which prediction difficulties may contribute to autism symptomatology and autistic traits.

### Limitations

Our study enhances current understanding of predictive language processing in autistic children. Nevertheless, this study has several limitations. First, regarding sample characteristics, our sample only included autistic children without language impairments, limiting the representativeness of the sample. Approximately 50% of autistic children demonstrate language impairments, with 30% being minimally verbal [69, 70], whereas our investigation included only autistic children without intellectual disability in the verbal domain (verbal IQ > 70). Future research should expand the sample range to include autistic children with diverse language abilities to better understand the heterogeneity of predictive language abilities in autism. This would require methodological adaptations for children with more limited comprehension skills. Building upon our current eye-tracking paradigm, potential adaptations could include using high-frequency vocabulary and simpler grammatical structures, providing longer visual preview periods to ensure stimulus familiarity, incorporating pre-training sessions to establish word-picture associations, reducing the number of distractors in visual arrays, and using more semantically constraining sentence contexts. These modifications would maintain the core focus on predictive processing during language comprehension while accommodating varying levels of linguistic competence.

Additionally, our study used the CARS-2 [34] and AQ-Child [35] to confirm or exclude autism diagnoses and measure symptom severity/characteristics, rather than gold-standard diagnostic instruments such as the Autism Diagnostic Observation Schedule (ADOS-2) [71] or the Autism Diagnostic Interview-Revised (ADI-R) [72]. While CARS-2 and AQ-Child provided systematic assessment of autism symptomatology/autistic traits, they may lack the granularity and diagnostic precision of comprehensive gold-standard evaluations. Future studies would benefit from using more detailed diagnostic assessments to ensure more precise characterization of participants’ autism profiles and to better understand how specific autism features may relate to predictive language processing patterns.

From a methodological perspective, our stimulus validation was conducted only with NT children, and we did not directly assess whether autistic children were equally familiar with all target words used in the task. While we controlled for this by matching groups on verbal IQ, and both groups demonstrated high and comparable accuracy in target identification during the experiment, direct validation with autistic participants or parental confirmation of word familiarity would have strengthened our methodology. Future studies should include stimulus validation specifically with autistic participants to ensure equal familiarity across groups. Additionally, while our study controlled for verbal IQ between groups, more subtle differences in real-time spoken word recognition between autistic and NT children, as was revealed in previous studies [32, 73, 74], could potentially influence the observed group differences in sentence-level predictive processing. Future research should incorporate specific measures of spoken word recognition speed and accuracy alongside sentence-level predictive processing tasks to more clearly separate these processes.

With respect to experimental design, the sentences employed in our task were limited to simple SVO-structured sentences. Linguistic structures in naturalistic contexts are far more complex and diverse. Future research could explore linguistic prediction in autistic children using more complex sentence structures, such as those with subordinate clauses, passive voice, or ambiguities [75], to examine how they predict more complex and varied linguistic input. Additionally, this study examined the performance of predictive processing in static, highly predictable contexts. However, naturalistic language environments are typically characterized by dynamic changes and uncertainties. Future research could explore the predictive adjustment abilities of autistic children when encountering changing contexts or situations that violate expectations, which might better reflect their language processing patterns in naturalistic communication contexts [3, 23].

Regarding the visual world paradigm itself, while this approach provides valuable insights into predictive language processing, we acknowledge its limitations in interpreting what participants are actually predicting during incremental sentence processing. Our paradigm constrains participants’ visual attention to a fixed set of four images, and we infer predictive processing from their looking patterns to these predetermined options. However, we cannot definitively determine the specific linguistic predictions participants generate or whether their eye movements reflect the precise words they expect to hear. Participants may predict alternative words not represented in our visual arrays, or their looking patterns may reflect broader semantic expectations rather than specific lexical predictions. Future research could employ complementary methodologies to provide more direct insight into participants’ predictions, such as cross-modal priming paradigms that do not constrain visual options [76], or EEG/ERP measures that can capture prediction error signals when expectations are violated [77, 78]. Additionally, computational modeling approaches, such as surprisal-based models or Bayesian updating frameworks [79- 82], could provide more detailed quantitative accounts of the prediction mechanisms underlying the observed eye movement patterns, potentially revealing whether group differences reflect distinct prediction strategies or varying precision in predictive processing.

Finally, concerning ecological validity, our study was conducted in controlled laboratory conditions with clear audio presentation, which may not reflect the complexity of real-world communication environments. Natural communication often occurs in noisy environments with competing auditory stimuli, which may pose additional challenges for predictive language processing [83]. This consideration is particularly relevant for autistic children, who commonly experience sensory processing difficulties and may show impaired speech perception in situations where there is background noise or concurrent speech [84]. Future research should investigate how environmental noise and sensory processing challenges interact with predictive language processing abilities in autism, as these factors may further impact prediction efficiency in naturalistic communication settings.

## Conclusions

In conclusion, this study provides novel insights into autistic children’s prediction efficiency and incremental processing strategies during spoken language comprehension. Our findings indicate that while both autistic and NT children employ a TRACE-like incremental processing strategy, autistic children exhibit reduced prediction efficiency compared to age- and language ability-matched NT peers. These findings enrich our understanding of language processing mechanisms in autism and provide practical implications for the intervention approaches targeting language development in autistic children.

## Supplementary Information

Below is the link to the electronic supplementary material.

Supplementary Material 1

## Data Availability

The datasets, analysis code, and materials supporting the findings of this study are openly available on the Open Science Framework (OSF) at https://doi.org/10.17605/OSF.IO/5W6ZT.

## Abbreviations

NT: Neurotypical
SVO: Subject-verb-object
AOI: Area of interest
CPA: Cluster-based permutation analysis
GCA: Growth curve analysis
DPA: Divergence point analysis
