# Supplementary material for: Prediction efficiency and incremental processing strategy during spoken language comprehension in autistic children: an eye-tracking study
Source: Mol Autism. 2025 Aug 4;16:39. doi: 10.1186/s13229-025-00674-0 (PMC12323288; doi:10.1186/s13229-025-00674-0)
Supplement: Supplementary file 1 — Supplementary Material 1 [file 13229_2025_674_MOESM1_ESM.docx]

**Table S1** Complete List of Sentence Stimuli

|  |  | Target image | Agent-related image | Action-related image | Unrelated image |
| --- | --- | --- | --- | --- | --- |
|  | The prince rides the horse. | horse | crown | bike | earrings |
| S1.1 | 王子 骑 马。 |  |  |  |  |
|  | The girl rides the bike. | bike | earrings | horse | crown |
| S1.2 | 女孩 骑 自行车。 |  |  |  |  |
|  | The prince wears the crown. | crown | horse | earrings | candy |
| S1.3 | 王子 戴 王冠。 |  |  |  |  |
|  | The girl wears the earrings. | earrings | bike | crown | horse |
| S1.4 | 女孩 戴 耳环。 |  |  |  |  |
|  | The bird builds the nest. | nest | worm | building | rice |
| S2.1 | 小鸟 建 鸟巢。 |  |  |  |  |
|  | The worker builds the building. | building | rice | nest | worm |
| S2.2 | 工人 建 楼房。 |  |  |  |  |
|  | The bird eats the worm. | worm | nest | rice | building |
| S2.3 | 小鸟 吃 虫子。 |  |  |  |  |
|  | The worker eats the rice. | rice | building | worm | nest |
| S2.4 | 工人 吃 米饭。 |  |  |  |  |
|  | The child drinks the milk. | milk | preschool | beer | factory |
| S3.1 | 小孩 喝 牛奶。 |  |  |  |  |
|  | The man drinks the beer. | beer | factory | milk | preschool |
| S3.2 | 男人 喝 啤酒。 |  |  |  |  |
|  | The child goes to the preschool. | preschool | milk | factory | beer |
| S3.3 | 小孩 去 幼儿园。 |  |  |  |  |
|  | The man goes to the factory. | factory | beer | preschool | milk |
| S3.4 | 男人 去 工厂。 |  |  |  |  |
|  | The cat catches the mouse. | mouse | couch | fly | lotus leaf |
| S4.1 | 猫咪 抓 老鼠。 |  |  |  |  |
|  | The frog catches the fly. | fly | lotus leaf | mouse | couch |
| S4.2 | 青蛙 抓 苍蝇。 |  |  |  |  |
|  | The cat jumps onto the couch. | couch | mouse | lotus leaf | fly |
| S4.3 | 猫咪 跳上 沙发。 |  |  |  |  |
|  | The frog jumps onto the lotus leaf. | lotus leaf | fly | couch | mouse |
| S4.4 | 青蛙 跳上 荷叶。 |  |  |  |  |
|  | The monkey lives in the forest. | forest | banana | ocean | fish |
| S5.1 | 猴子 住在 森林。 |  |  |  |  |
|  | The shark lives in the ocean. | ocean | fish | forest | banana |
| S5.2 | 鲨鱼 住在 海洋。 |  |  |  |  |
|  | The monkey eats the banana. | banana | forest | fish | ocean |
| S5.3 | 猴子 吃 香蕉。 |  |  |  |  |
|  | The shark eats the fish. | fish | ocean | banana | forest |
| S5.4 | 鲨鱼 吃 鱼。 |  |  |  |  |
|  | Mom finds the necklace. | necklace | bed | egg | coop |
| S6.1 | 妈妈 找到 项链。 |  |  |  |  |
|  | The hen finds the egg. | egg | coop | necklace | bed |
| S6.2 | 母鸡 找到 鸡蛋。 |  |  |  |  |
|  | Mom sleeps in the bed. | bed | necklace | coop | egg |
| S6.3 | 妈妈 睡在 床上。 |  |  |  |  |
|  | The hen sleeps in the coop. | coop | egg | bed | necklace |
| S6.4 | 母鸡 睡在 窝里。 |  |  |  |  |

**S2. GCA Model Selection Process**

To determine the optimal GCA model for capturing the trajectory of log-gaze proportion ratios (target vs. unrelated distractor), we fitted a series of nested models with increasing polynomial complexity, starting with a linear polynomial linear mixed-effect model and progressively adding higher-order time terms (up to quartic). Each model included the specified orthogonal time polynomials interacting with group as fixed effects, and incorporated random intercepts for participants and trials. Models were compared using ANOVA to determine whether the addition of higher-order polynomials significantly improved model fit, with model parsimony taken into account through the Akaike Information Criterion (AIC) and the Bayesian Information Criterion (BIC) values.

Results of the model comparison are presented in Table S2. The linear polynomial model served as our baseline. ANOVA results indicated that adding a quadratic term significantly improved model fit (χ²(2) = 966.57, *p* < .001), indicating substantial non-linearity in the log-ratio trajectories. The addition of a cubic term further improved model fit (χ²(2) = 71.99, *p* < .001), as did the addition of a quartic term (χ²(2) = 29.80, *p* < .001). The addition of a fifth-order polynomial (quintic term) resulted in a statistically significant improvement (χ²(2) = 7.72, *p* = .021), but the magnitude of improvement was relatively small compared to previous polynomial additions.

Although the quintic model showed the lowest AIC value (308767), the difference from the quartic model was minimal (ΔAIC = 4). The BIC, which more strongly penalizes model complexity, favored the quartic model (308903) over the quintic model (308920). Considering the principle of parsimony and the relatively modest improvement gained by adding the quintic term, we selected the quartic model, which included linear, quadratic, cubic, and quartic time terms interacting with group, as our final GCA model for analysis of the log-gaze proportion ratio trajectories.

**Table S2** Growth Curve LMM Model Comparison Results

| **Model** | **AIC** | **BIC** | **χ²** | **df** | ***p*** | ***R*² (Marginal)** | ***R*² (Conditional)** |
| --- | --- | --- | --- | --- | --- | --- | --- |
| Linear | 309827 | 309898 | - | - | - | .0573 | .0781 |
| Quadratic | 308865 | 308956 | 966.57 | 2 | < .001 | .0620 | .0832 |
| Cubic | 308797 | 308908 | 71.99 | 2 | < .001 | .0624 | .0835 |
| Quartic | 308771 | 308903 | 29.80 | 2 | < .001 | .0625 | .0836 |
| Quintic | 308767 | 308920 | 7.72 | 2 | .021 | .0625 | .0836 |

LMM = linear mixed-effect models. AIC = Akaike Information Criterion. BIC = Bayesian Information Criterion.

**S3. GCA with Verbal IQ Covariate**

To address potential confounding effects of verbal IQ, we conducted an additional GCA including verbal IQ as a covariate. The inclusion of verbal IQ as a covariate did not substantially alter the key findings. The critical group effects remained significant after controlling for verbal IQ: The main effect of group (β = -0.02, *SE* = 0.01, *p* = .001) and the group × linear time interaction (β = -0.10, *SE* = 0.01, *p* < .001) were both significant and of similar magnitude to the original model. The group × cubic interaction remained marginally significant (β = 0.02, *SE* = 0.01, *p* = .045). These results confirm that the observed group differences in predictive fixation trajectories are not attributable to verbal IQ differences between groups. See Table S3 for GCA model statistics.

**Table S3** GCA Results With Verbal IQ as Covariate

| Fixed Effects | *B* | *SE* | *t* | *p* |
| --- | --- | --- | --- | --- |
| (Intercept) | 0.01 | 0.04 | 0.149 | .881 |
| Linear | 0.95 | 0.01 | 104.96 | **< .001** |
| Quadratic | 0.28 | 0.01 | 30.90 | **< .001** |
| Cubic | -0.07 | 0.01 | -7.95 | **< .001** |
| Quartic | -0.05 | 0.01 | -5.35 | **< .001** |
| Group | -0.02 | 0.01 | -3.33 | **.001** |
| Verbal IQ | 0.001 | 0.0004 | 3.07 | **.003** |
| Linear × Group | -0.10 | 0.01 | -11.26 | **< .001** |
| Quadratic × Group | 0.01 | 0.01 | 1.29 | .199 |
| Cubic × Group | 0.02 | 0.01 | 2.00 | **.045** |
| Quartic × Group | 0.004 | 0.01 | 0.47 | .641 |

GCA = growth curve analysis. *B* = unstandardized coefficient. *SE* = standard error. Linear, Quadratic, Cubic, Quartic, and Quintic refer to orthogonal polynomial time terms. NT children served as the reference group. Significant *p*-values are shown in bold.

**S4. AQ Subscale Correlations With Prediction Efficiency**

We examined correlations between the five AQ-Child subscales (social skills, attention switching, attention to detail, communication, and imagination) (1) and both prediction efficiency measures. Higher scores on each AQ subscale indicate greater autism-related difficulties in that specific domain. To control for multiple comparisons across the five subscales within each group, we applied false discovery rate (FDR) correction using the Benjamini-Hochberg method (2).

Tables S4 and S5 present detailed correlation analyses for all AQ subscales with both prediction efficiency measures. Figures S1 and S2 provides visual representations of these relationships through scatter plots for each AQ dimension. The most robust finding across both groups was the significant association between prediction efficiency and communication difficulties. After FDR correction, communication subscale scores remained significantly correlated with both prediction measures in both groups, with effect sizes ranging from moderate to large (|*r*| = .41-.51). Specifically, communication scores showed negative correlations with log-ratio scores in both the autistic group (*r* = -.45, FDR-*p* = .012) and NT group (*r* = -.41, FDR-*p* = .012), and positive correlations with divergence points in both the autistic group (*r* = .49, FDR-*p* = .009) and NT group (*r* = .51, FDR-*p* < .001), indicating that reduced prediction efficiency was consistently associated with increased autism-related communication difficulties across groups.

Beyond communication scores, in the autistic group, both social skills scores (*r* = -.34, FDR-*p* = .040) and attention switching scores (*r* = -.35, FDR-*p* = .040) were significantly correlated with log-ratio scores, and attention switching scores also showed significant associations with divergence points (*r* = .48, FDR-*p* = .009), indicating that reduced prediction efficiency was associated with difficulties in these areas.

In the NT group, beyond communication scores, social skills scores were significantly associated with divergence points (*r* = .38, FDR-*p* = .010), and imagination scores also showed significant associations with divergence points (*r* = .39, FDR-*p* = .010), indicating that even within the neurotypical population, delayed prediction timing was associated with multiple aspects of autism-related social and cognitive difficulties.

**Table S4** Correlations Between AQ Subscales and Log-ratio Scores

| AQ Subscales | Autistic Group (*N* = 45) | | | |  | NT Group (*N* = 52) | | | |
| --- | --- | --- | --- | --- | --- | --- | --- | --- | --- |
|  | *r* | *p* | FDR-*p* | 95% CI |  | *r* | *p* | FDR-*p* | 95% CI |
| Social Skills | -.340 | .024^*^ | .040^*^ | [-.579, -.048] |  | -.291 | .036^*^ | .061 | [-.522, -.020] |
| Attention Switching | -.352 | .019^*^ | .040^*^ | [-.587, -.061] |  | .114 | .422 | .422 | [-.164, .375] |
| Attention to Detail | -.003 | .986 | .986 | [-.299, .294] |  | .238 | .089 | .111 | [-.037, .480] |
| Communication | -.445 | .002^**^ | .012^*^ | [-.655, -.171] |  | -.411 | .002^**^ | .012^*^ | [-.615, -.155] |
| Imagination | -.162 | .294 | .368 | [-.438, .142] |  | -.295 | .034^*^ | .061 | [-.525, -.024] |

^*^ *p* < .05, ^**^ *p* < .01. FDR-*p* = FDR-corrected *p*-values for multiple comparisons across the five subscales within each group.

**Table S5** Correlations Between AQ Subscales and Divergence Points

| AQ Subscales | Autistic Group (*N* = 45) | | | |  | NT Group (*N* = 52) | | | |
| --- | --- | --- | --- | --- | --- | --- | --- | --- | --- |
|  | *r* | *p* | FDR-*p* | 95% CI |  | *r* | *p* | FDR-*p* | 95% CI |
| Social Skills | .297 | .084 | .139 | [-.041, .573] |  | .383 | .006^**^ | .010^*^ | [.117, .598] |
| Attention Switching | .480 | .004^**^ | .009^**^ | [.175, .701] |  | -.076 | .598 | .598 | [-.347, .206] |
| Attention to Detail | .035 | .843 | .843 | [-.302, .364] |  | -.199 | .165 | .207 | [-.452, .084] |
| Communication | .495 | .003^**^ | .009^**^ | [.194, .711] |  | .513 | < .001^***^ | < .001^***^ | [.274, .693] |
| Imagination | .173 | .321 | .402 | [-.170, .478] |  | .389 | .005^**^ | .010^*^ | [.124, .602] |

^*^ *p* < .05, ^**^ *p* < .01, ^***^ *p* < .001. FDR-*p* = FDR-corrected *p*-values for multiple comparisons across the five subscales within each group.


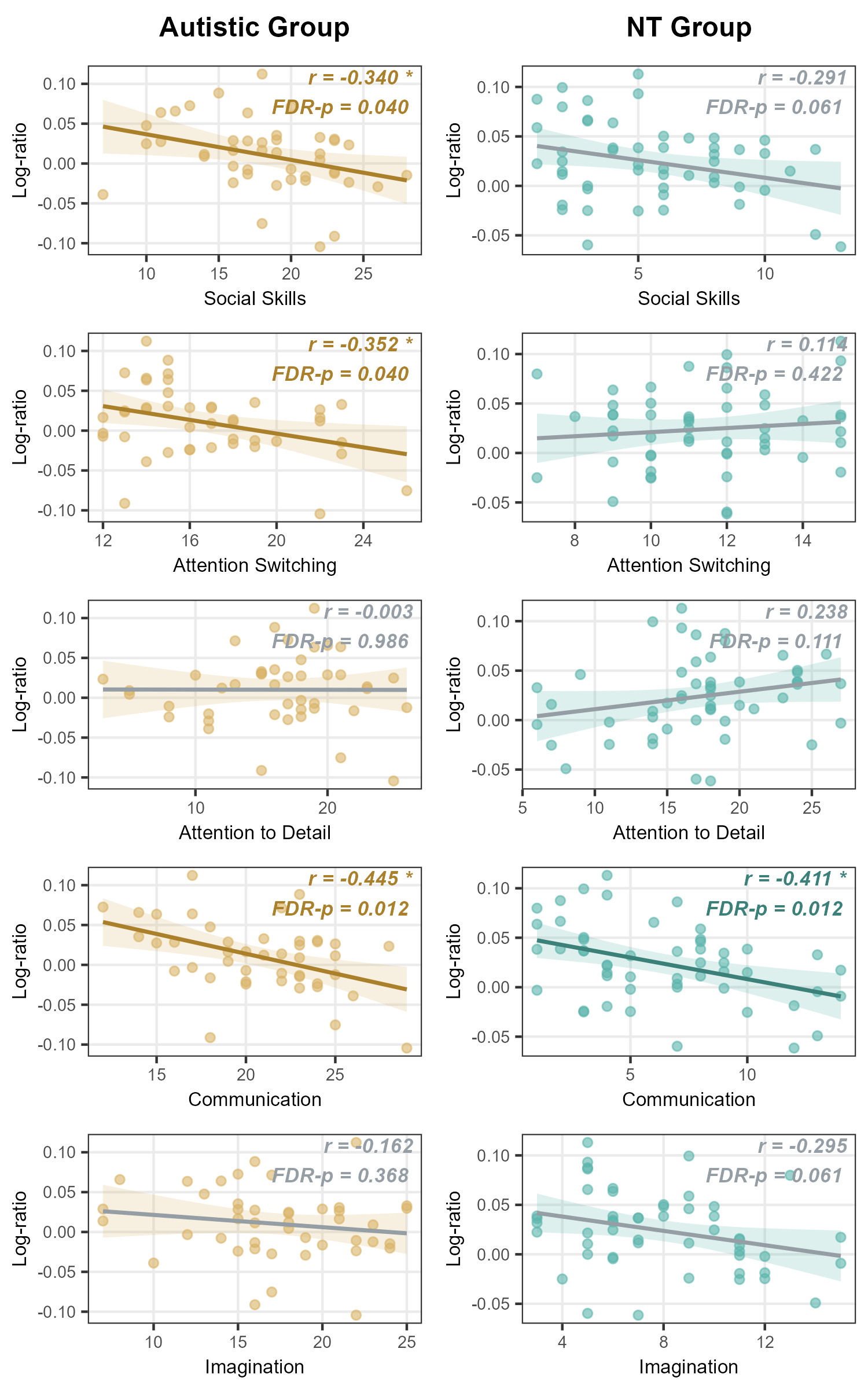


**Fig. S1** Correlations between AQ subscales and log-ratio scores

^*^ indicates significance after FDR correction.


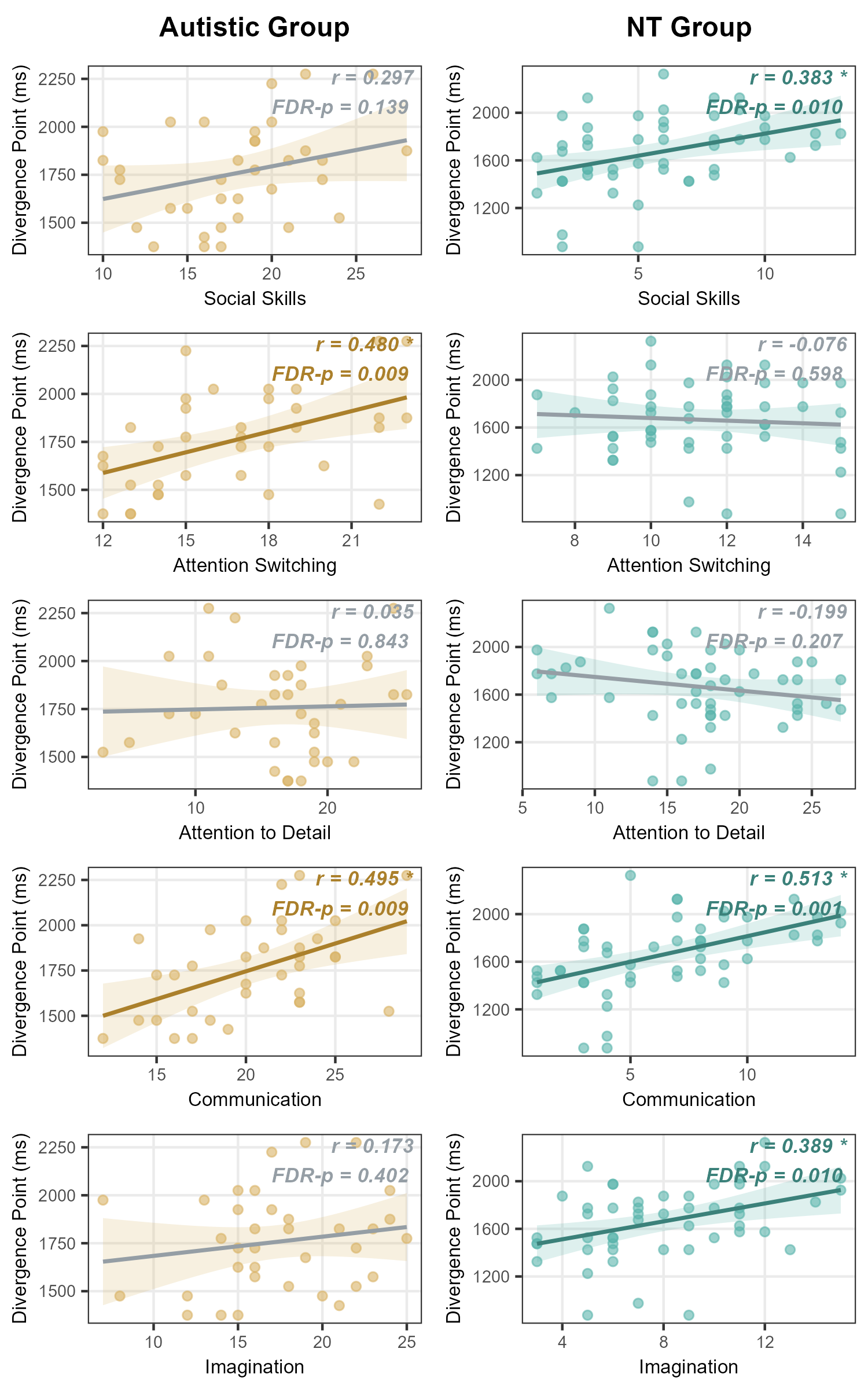


**Fig. S2** Correlations between AQ subscales and divergence points

^*^ indicates significance after FDR correction.

**References**

1. Auyeung B, Baron-Cohen S, Wheelwright S, Allison C. The Autism Spectrum Quotient: Children’s Version (AQ-Child). J Autism Dev Disord. 2008;38(7):1230-40.

2. Benjamini Y, Hochberg Y. Controlling the False Discovery Rate: A Practical and Powerful Approach to Multiple Testing. Journal of the Royal Statistical Society: Series B (Methodological). 1995;57(1):289-300.
